# Nano Carbon-Based Hybrid Strategies for Mitigating Silicon Anode Expansion in Lithium-Ion Batteries: A Comprehensive Review

**DOI:** 10.3390/ma18245532

**Published:** 2025-12-09

**Authors:** Wonhwa Lee, Yunki Jung, Jin-Yong Hong, Young-Pyo Jeon, Jea Uk Lee

**Affiliations:** 1 Department of Advanced Materials Engineering for Information and Electronics, Kyung Hee University, 1732 Deogyeong-daero, Giheung-gu, Yongin-si 17104, Republic of Korea; lwh980304@khu.ac.kr (W.L.); yunki0930@khu.ac.kr (Y.J.); 2 Hydrogen & C1 Gas Research Center, Korea Research Institute of Chemical Technology (KRICT), 141 Gajeong-ro, Yuseong-gu, Daejeon-si 34114, Republic of Korea; jyhong@krict.re.kr; 3 Advanced Materials and Chemical Engineering, University of Science and Technology (UST), 217 Gajeong-ro, Yuseong-gu, Daejeon-si 34113, Republic of Korea

**Keywords:** lithium-ion batteries, silicon, carbon nanotubes, graphene, carbon nanofibers

## Abstract

Silicon is considered one of the most promising anode materials for lithium-ion batteries because of its high theoretical capacity and low lithiation potential. However, its practical application is limited by significant volume expansion, unstable solid–electrolyte interphase formation, and poor intrinsic conductivity. This review summarizes recent advances in hybrid strategies using multi-walled carbon nanotubes (MWCNTs), single-walled carbon nanotubes (SWCNTs), graphene, carbon nanofibers (CNFs), and pitch-derived carbons. We compare their respective benefits and drawbacks regarding conductivity, structural resilience, and scalability, while also addressing critical challenges such as dispersion, defect control, and processing costs. The discussion emphasizes the importance of hierarchical, multifunctional architectures that combine different forms of carbon to achieve synergistic performance. Finally, we outline future directions in interfacial engineering, defect and doping optimization, and electrode design under high-loading conditions. We believe that this review can offer perspectives on developing durable, energy-dense, and commercially viable silicon anodes for next-generation lithium-ion batteries.

## 1. Introduction

The demand for high-performance lithium-ion batteries (LIBs) has risen sharply with the growth of electric vehicles, portable electronics, and large-scale energy storage systems. Graphite is the most commonly used anode material in commercial LIBs due to its low cost and high electrical conductivity (~3 × 10^5^ S/m) [1]. However, a graphite anode has a low theoretical specific capacity (372 mAh/g for LiC_6_) and limited rate capability because of sluggish Li^+^ transport within its layered structure. So far, several materials such as germanium (Ge), phosphorus (P), silicon (Si), and tin (Sn) have been investigated as anodes for LIBs because of their high specific capacities [2,3]. Among these, silicon (Si) has garnered significant interest due to its remarkable theoretical capacity (~4200 mAh/g), low lithiation potential, and natural abundance [4,5]. Nonetheless, the commercialization of Si anodes faces major hurdles, including severe volume expansion (~300–400%) during lithiation and delithiation, poor electrical conductivity, and the continual formation of an unstable solid–electrolyte interphase (SEI). These issues collectively cause rapid capacity loss and poor cycle life [6,7]. Addressing these fundamental challenges requires designing strategies to stabilize Si anodes without sacrificing their energy density.

Over the past decade, various methods have been proposed to address silicon’s (Si) limitations. Nano structuring, such as nanoparticles, nanowires, and nanosheets, reduces pulverization by decreasing the diffusion length and stress but can increase surface area, leading to side reactions and lowering the initial Coulombic efficiency (ICE) [8]. Composite engineering, especially with carbon materials, improves conductivity and strength. One-dimensional carbon nanotubes (CNTs) and two-dimensional coatings like graphene or amorphous carbon form conductive frameworks to maintain electronic pathways and buffer stress [9,10]. Polymeric coatings—including natural and conductive polymers—also help suppress side reactions, regulate SEI formation, and provide flexibility for Si expansion [11]. These strategies emphasize the value of synergistic architectures combining Si with conductive and protective matrices. Recent reviews note that moving from lab to industry requires more than incremental improvements [12]. For micron-sized Si, structural optimization is critical. Effective designs such as yolk–shell, void-engineered, or hierarchical composites balance stability, transport, and ease of manufacturing [13]. Innovations such as artificial SEI, prelithiation, and improvements to binders are key to enhancing ICE system-level performance and cycling stability. Ultimately, future Si anodes will rely on integrated multiscale design, combining nanoscale control with scalable synthesis and electrode engineering.

**Figure 1:**
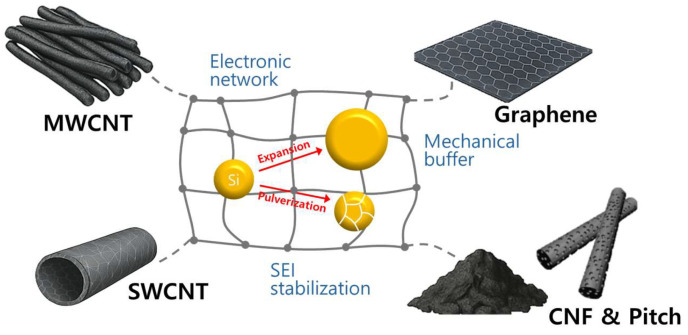
Challenges and strategies for structurally stable Si anodes using MWCNT, SWCNT, graphene, CNF, and pitch-derived carbon.

In this review, we focus on recent progress in hybrid strategies that utilize multi-walled carbon nanotubes (MWCNTs), single-walled carbon nanotubes (SWCNTs), graphene, carbon nanofibers (CNFs), and pitch-derived carbons to mitigate the significant volume expansion of Si anodes for Li-ion batteries (Figure 1). Table 1 provides a detailed comparison of various carbon materials, highlighting differences in electrical conductivity, specific surface area, defect content, cost, processability, and synthesis-related factors, including the number of steps, total energy consumption, precursor cost, and scalability [14,15,16,17]. Special emphasis is placed on comparing the intrinsic advantages and limitations of each carbon material, including their roles in enhancing electronic conductivity, providing mechanical buffering, and stabilizing interfacial chemistry. We aim to include and present recent research studies published since 2020 as the primary content of our review, focusing on the synthesis and fabrication of silicon–carbon architectures. Additionally, we discuss how these carbon-based hybrids can be intentionally designed to address ongoing challenges such as unstable SEI formation, low initial Coulombic efficiency, and limited electrode density.

To enhance readability and searchability, we organized each highlighted study into sections: the first introduces the author, year, and key findings; the second describes the hybrid carbon material’s morphology and properties used to reduce silicon expansion; the third explains the process for coating silicon particles with carbon nanomaterials; the fourth lists key battery metrics like capacity, cycle stability, and rate capability; the final summarizes other notable features, innovations, and significance. Each section ends with a table summarizing parameters such as initial capacity, efficiency, electrode loading, stability, retention, and C-rate for quick comparison. We also discuss future improvements and development directions to scale up these hybrid systems for commercial viability as next-generation silicon battery anodes.

## 2. Applications of Nano Carbons

### 2.1. MWCNT Strategies for Structurally Stable Si Anodes

Despite their very high theoretical capacity, Si-based anodes face severe structural degradation due to approximately 300% volume expansion during lithiation and delithiation. This causes particle pulverization, loss of electrical contact, and continuous SEI reformation [39]. To overcome these issues, recent research has focused on incorporating MWCNTs as both conductive frameworks and mechanical buffers to create robust composite structures [40,41]. Thanks to their high electrical conductivity and mechanical flexibility, MWCNTs have been integrated with nano-or micron-sized Si particles in various configurations to build three-dimensional electron-conducting networks. These networks help accommodate volume changes, preserve interfacial stability, and improve ion and electron transport [42,43,44]. Importantly, beyond serving as passive conductive additives, MWCNTs embedded in or chemically bonded to the active material have shown improved performance by establishing site-specific, percolating electron pathways. These pathways are essential for developing high-performance Si anodes [45]. This section systematically reviews the structural configurations, synthesis methods, electrochemical behaviors, and practical implications of recent MWCNT–Si composites for advanced LIBs.

A magnesiothermic reduction method was developed to create uniform core–shell MWCNT@Si nanocomposites for high-performance LIB anodes. It uses the CNT scaffold to reduce Si volume expansion. The structure features 30–50 nm MWCNT backbones with a porous Si shell of 5–15 nm crystallites. The composite has a large BET surface area (255 m^2^ g^−1^) and ~40 nm mesopores for strain relief and electronic pathways. Fabrication involved HNO_3_-steam functionalization of MWCNTs, Stöber TEOS hydrolysis to grow MWCNT@SiO_2_ nanocables, and magnesiothermic reduction at 650 °C, followed by acid leaching to form crystalline Si shells around CNT cores. Electrochemically, it shows first-cycle capacities of 1547/786 mAh g^−1^ (initial Coulombic efficiency ~51%), exceeding 95% after five cycles. It retains about 800 mAh g^−1^ (10 cycles) and 520 mAh g^−1^ (70 cycles) at 400 mA g^−1^, outperforming bulk Si and bare MWCNT. Rate testing yields 900, 734, 650, and 536 mAh g^−1^ at 0.2, 0.4, 0.8, and 1.6 A g^−1^, with 680 mAh g^−1^ at return to 0.4 A g^−1^. EIS shows better conductivity than bare Si. Increasing CNT to 40 wt% improves electron network, reduces Si aggregation, and enhances stability. However, low initial Coulombic efficiency and limited long-term retention highlight the need for pre-lithiation and coatings for SEI formation.

Si–multi-walled carbon nanotube–carbon (Si–MWCNTs–C) microspheres employing a CNT-supported spherical scaffold were reported to mitigate Si’s volume expansion and improve LIB anode performance [40]. The microspheres, several micrometers in diameter, encapsulated around 100 nm Si nanoparticles within a porous MWCNTs/C matrix, forming a continuous 3D conductive network that prevented Si expansion during cycling. The microspheres were created by wet ball milling a 2:1 Si/MWCNTs slurry, blending with PEG and carbonizing, or spray drying a Si/MWCNTs–glucose dispersion followed by argon pyrolysis; both methods seal Si within a carbon-rich shell. Electrochemically, the composite delivered first-cycle discharge/charge capacities of 1848/1228 mAh g^−1^ (initial Coulombic efficiency 66.4%), retained approximately 1050 mAh g^−1^ after 60 cycles at 0.2 A g^−1^, and still provided 415 mAh g^−1^ at 6 A g^−1^; the average capacity at 0.2 A g^−1^ was about 1100 mAh g^−1^. Ex situ SEM observations reported in the literature showed minimal cracking after 100 cycles, while impedance measurements indicated a stabilized charge-transfer resistance (Rct) of approximately 41 Ω, both attributed to the robust 3D network and carbon bridges that maintained electrical integrity throughout cycling. Overall, the strengths of this work lie in the structural novelty of a CNT-supported spherical scaffold that maintained a continuous 3D percolation network, along with straightforward synthesis methods (wet ball milling/carbonization or spray drying/pyrolysis). Despite these advantages, the initial Coulombic efficiency (66.4%) remains suboptimal for direct LIB applications.

A binder- and current-collector-free flexible anode was created by depositing about 0.37 µm of amorphous Si thin film onto oxidized MWCNT buckypaper, resulting in a self-supporting electrode that can be rolled or bent without cracking [41]. The woven MWCNT network offered a highly conductive (1.6 × 10^4^ S m^−1^) yet mechanically flexible scaffold that accommodated Si volume changes, while its dense, heat-treated microporous texture further buffered expansion and stabilized the SEI layer. Fabrication included acid–oxidative filtration to produce 17-µm-thick buckypaper, vacuum heat treatment at 600 °C, DC magnetron sputtering of Si (200 W, 15 min), and a final post-anneal at 600 °C for 1 h, creating “HSi_HBP” electrodes; a non-annealed version (“USi_HBP”) served as the control. HSi_HBP exhibited first-cycle discharge/charge capacities of 1675/194 µAh (initial Coulombic efficiency ≈ 12%) and maintained 193/192 µAh after 100 cycles at 125 µA (99.5% capacity retention), significantly outperforming USi_HBP (108/106 µAh, 41% retention). Remarkably, after 5000 bends at a radius of 8.3 mm, the electrode still delivered 234/206 µAh with only about 15% capacity loss and at least 88% efficiency, highlighting its potential for flexible or wearable Li-ion batteries. However, the very low initial Coulombic efficiency (~12%) and the inherently low areal capacity due to the ultrathin (~0.37 µm) Si layer and high CNT fraction limit immediate practical use.

A three-component Si anode, where nano-Si is embedded with inert nano-Ag and wrapped by a multilayer porous carbon nanosheet/MWCNT matrix (Si/Ag/PM), was developed and demonstrated significantly improved electrochemical performance in LIBs [42]. To reduce Si’s volume expansion, approximately 100 nm Si particles and 30 nm Ag nanoparticles were uniformly anchored within a 3D, CNT-reinforced porous carbon scaffold; this interconnected layered network created continuous electron/ion pathways while providing internal voids that absorb mechanical stress during cycling. The composite was made by reducing Ag^+^ onto dispersed Si using a glucose-assisted water bath, followed by calcination at 800 °C to produce Si/Ag; ball milling sodium citrate with MWCNTs and carbonizing at 850 °C to create conductive PM; then slurry mixing Si/Ag with PM, drying, and a second argon calcination at 800 °C to form the final Si/Ag/PM spheres. Electrochemically, the anode shows a high initial Coulombic efficiency of 78.9% at 0.1 A g^−1^ and maintains 706 mAh g^−1^ after 300 cycles at 1 A g^−1^ (68.6% retention). The rate capability reaches 1651, 1135, and 559 mAh g^−1^ at 0.1, 0.5, and 3 A g^−1^, respectively, with full capacity recovery when the current is lowered again. Besides improving conductivity, the authors emphasize that the Ag-decorated PM framework accelerates Si lithiation/delithiation, suppresses SEI re-formation, and can be produced through an environmentally friendly process suitable for industrial use.

A freeze-drying–assisted method that combined Si nanoparticles with MWCNTs and then encapsulated them in a porous-carbon (FPC) skeleton derived from flour plus a sucrose-derived carbon (SC) overcoat produced the Si–MWCNTs–PVPC–FPC–SC-1 composite for high-rate LIBs [43]. The material features 40–60 nm Si and MWCNTs uniformly embedded within a 3D FPC host (BET ≈ 1850 m^2^ g^−1^; mean pore size ≈ 3.9 nm), with several CNTs piercing the thin SC shell, creating a hierarchical conductive/void network that prevents Si volume expansion while maintaining electronic continuity. The synthesis involved PVP-assisted ultrasonication and shear mixing of Si/MWCNTs, freeze-drying at −50 °C for 12 h, carbonization to form Si–MWCNTs–PVPC, electrostatic adsorption into FPC, sucrose coating, and a second pyrolysis—steps that together greatly reduce agglomeration compared to oven blast-drying controls. Electrochemically, the composite delivered 1347.5 mAh g^−1^ when the current was returned to 0.1 A g^−1^ after a 5 A g^−1^ rate test and still maintained 501 mAh g^−1^ after 500 cycles at 1 A g^−1^ with over 99.5% capacity retention; EIS revealed a charge-transfer resistance of 36.97 Ω and a Li^+^ diffusion coefficient of 2.06 × 10^−15^ cm^2^ s^−1^, both superior to porous-carbon controls. Notably, freeze-drying reduced the native SiO_2_ shell to approximately 3.1 nm (compared with 9.3 nm after blast drying) and increased Si–O–C interfacial bonding to 11.8%, resulting in excellent retention at a relatively high loading level (2.0 mg cm^−2^).

A comparison of single versus hybrid conductive additives in SiO_2_/Si/PAN composite anodes identified a 1:1 mixture of reduced graphene oxide (rGO) and MWCNTs—denoted M/r_1:1—as optimal, delivering superior capacity and stability over either additive alone [44]. To accommodate Si’s expansion, hollow approximately 400 nm SiO_2_ spheres loaded with nanocrystalline Si were embedded within a PAN-derived carbon matrix, while interleaved 2D rGO sheets and 1D CNT “bridges” construct a porous, continuous 3D network that cushions stress and maintains conductivity. The scalable fabrication combined emulsion-templated SiO_2_ cores, liquid-phase mixing of Si/PAN followed by carbonization at 800 °C, and conventional slurry casting with 10 wt% of the tailored carbon additive to form flexible electrodes. Electrochemically, M/r_1:1 delivered an initial capacity of 3009 mAh g^−1^ with a 96.0% initial Coulombic efficiency, retained 1513 mAh g^−1^ after 100 cycles at 0.1 C, sustains 1627, 1133, and 753 mAh g^−1^ at 0.2, 0.5, and 1 C, respectively, and exhibits a low charge-transfer resistance of 129.6 Ω. These results are attributed to the rGO–CNT bridge network, which shortens electron/ion pathways and reduces internal resistance without excessive carbon loading.

**Figure 2:**
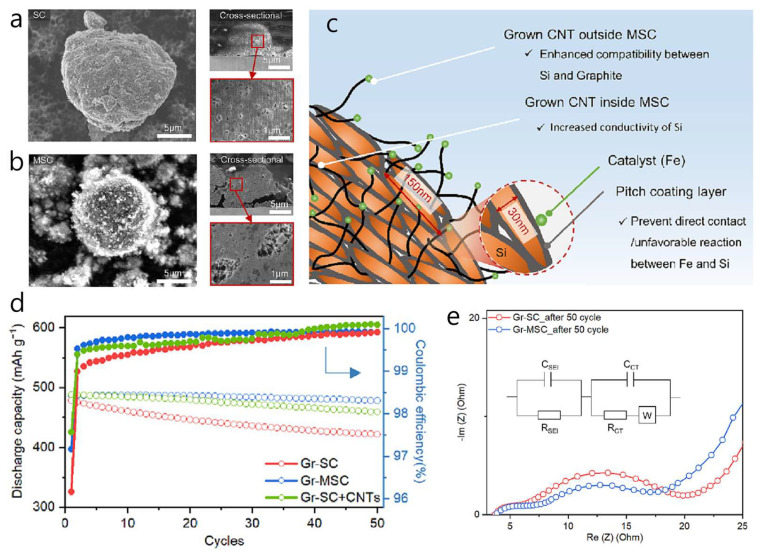
SEM images of SC (**a**) and MSC (**b**). (**c**) Cross-sectional schematic view showing the detailed elucidated mechanism of additional electric network on MSC. (**d**) Cycling performance and Coulombic efficiency of each blended electrode for 50 cycles under 0.5 C. (**e**) Nyquist plots for Gr-SC and Gr-MSC after 50 cycles. (Reproduced with permission from [45], copyright 2023, Elsevier).

A Si composite with MWCNTs (MSC), acting as an in situ electrical bridge within commercial graphite-blended anodes, was shown to significantly enhance electrical connectivity between graphite and micron-scale Si particles during cycling [45]. Si expansion was limited by wrapping 146 nm Si flakes in a pitch-derived carbon shell and growing about 15 wt% MWCNTs from the shell’s surface. This one-dimensional CNT network offered high conductivity, while the dense carbon/graphite framework buffered Si expansion (Figure 2a–c). MWCNTs grew via catalytic CVD: Fe(NO_3_)_3_ was evenly dispersed on carbon-coated Si particles, reduced at 700 °C in H_2_, then exposed to C_2_H_4_ to form CNTs that anchored at the Si/graphite interface during slurry processing. In electrochemical tests, a Gr–MSC half-cell had an initial specific capacity of 453 mAh g^−1^ with an initial Coulombic efficiency of 80%, retaining 98% after 50 cycles at 0.5 C (Figure 2d). Rate performance reached 88% of the 5 C capacity at 5 C, and four-point probe resistivity fell by 18% compared to a CNT-free control. In full cells (NCM 622 cathode, N/P 05), the Gr–MSC anode achieved 88% ICE and 90% capacity retention over 200 cycles, with an average charge plateau of 86 V. The lower Rct of Gr–MSC indicated faster interfacial charge transfer than Gr–SC, likely because the MWCNT network maintained robust electrical pathways during extended cycling (Figure 2e). Notably, the study highlights that externally added CNTs cannot match the effectiveness of CNTs chemically grafted onto the Si surface, underscoring the importance of site-specific conductive networks when combining alloy-type Si with intercalation-type graphite for high-energy LIBs. The innovative aspect of this work is the dual use of pitch as both a conformal coating on silicon and as a growth-compatible matrix for MWCNTs, which suppressed Si volume expansion and reduced interfacial resistance between Si and graphite during slurry processing.

A flexible, standalone Si/MWCNT tissue was created by uniformly sputter-coating commercial MWCNT buckypaper with an approximately 427 nm amorphous Si shell. This forms an anode that combines the flexibility of CNT fabrics with the high capacity of Si [46]. The macroscopic 3D CNT web serves as an elastic current collector and mechanical scaffold, while the conformal Si layer provides Li-ion storage sites. The continuous CNT network ensures rapid electron and ion transport, and additional voids reduce SEI growth. RF magnetron sputtering deposited 0.5–2.0 mg/cm^2^ of Si onto the roughly 100 µm-thick CNT tissue in a single vapor-phase step, resulting in a binder- and copper-collector–free electrode that can be rolled, bent, or punched directly into coin-cell discs. Chemically, the optimized 1.0 mg/cm^2^ Si/MWCNT tissue delivered an initial discharge of approximately 1300 mAh/g, reached 94% Coulombic efficiency by the 10th formation cycle, and stabilized at 290 mAh/g after 40 cycles (compared to 109 mAh/g for bare CNTs). The key strength of this manufacturing process is its use of a simple sputtering technique to create a binder- and current-collector–free Si anode. This method offers a promising pathway toward ultralightweight, flexible lithium-ion batteries.

**Figure 3:**
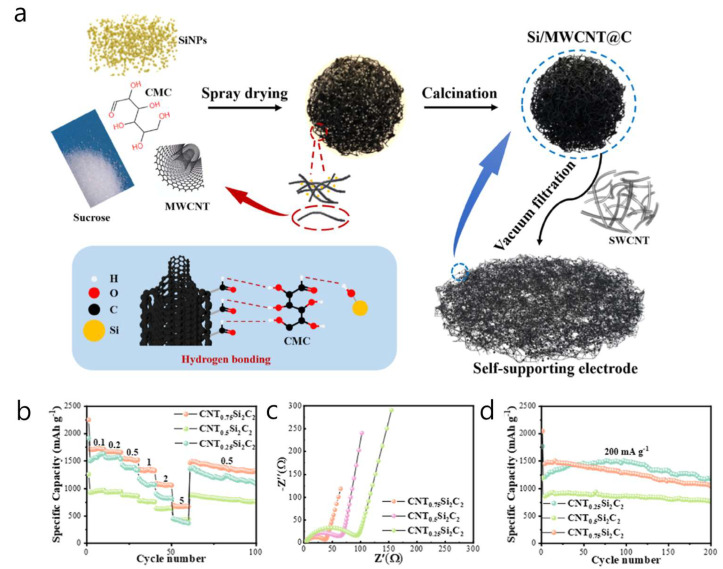
(**a**) Preparation of Si/MWCNT@C and Self-supporting electrode. (**b**) Rate capabilities, (**c**) Nyquist plots, and (**d**) cycling performance of Si/MWCNT@C anode. (Reproduced with permission from [47], copyright 2024, American Chemical Society).

A spray-dried Si/MWCNT@C microsphere structure was created, where approximately 90 nm Si nanoparticles were intertwined with about 15 wt% MWCNTs and enclosed in a sucrose-derived carbon matrix. This formed a self-supported 3D conductive network that connects neighboring Si islands and prevents electrical isolation during cycling [47]. The porous, roughly 5 µm secondary particles featured a radially percolating CNT scaffold that conducted electrons. At the same time, the dual-carbon framework (composed of in situ pyrolyzed sucrose and graphitic CNT walls) accommodated the volumetric expansion of Si and limited SEI growth. Synthesis included ultrasonic dispersion of Si, CNTs, and sucrose in water, rotary-spray-drying of the slurry to produce spherical precursors, and a single-step carbonization at 800 °C in Ar, resulting in binder-free powders that could be slurry-cast with less than 2 wt% additional carbon (Figure 3a). A Li∥CNT_0.5_Si_2_C_2_ half-cell (0.5 A g^−1^) initially delivered a reversible capacity of 1466 mAh g^−1^ with 83% ICE, and still provided 536.6 mAh g^−1^ after 300 cycles at 1 A g^−1^ (80.5% retention). The rate capability maintained 62% of the capacity at 0.5 A g^−1^, and charge-transfer resistance was 34% lower than that of a control without CNTs (Figure 3b–d). In a full cell (LiFePO_4_ cathode, N/P ≈ 1.1), the composite anode initially delivered 72.4 mAh g^−1^ and retained 79.8% after 400 cycles, with over 99% Coulombic efficiency. A key strength of this study is the use of a cost-effective, mass-producible spray-drying process to produce porous spherical Si/MWCNT@C anodes; embedding MWCNTs during spray drying created a continuous, through-particle percolation network that maintained electrical transport within both the microspheres and the self-supported electrodes.

A defect-engineered and oxygen-functionalized MWCNT architecture was developed, achieving dual improvements in Li^+^ storage capacity and rate kinetics by integrating multiple reversible reaction sites into a single carbon framework [48]. The authors increased Li-ion uptake by introducing nanopores, open ends, and oxygen-rich groups (C–O, C=O, and O–C=O) through acid etching and thermal treatment, transforming smooth, inert MWCNTs into a porous hybrid of graphitic and amorphous domains with significantly higher defect density. These structural changes enabled multiple storage mechanisms: Li^+^ intercalation (<0.4 V), hollow-core filling (~0.6 V), defect/nanopore adsorption (0.6–1.5 V), and redox reactions with surface oxygen groups (1.5–3.0 V), as shown by cyclic voltammetry and b-value analysis (b ≈ 0.8–0.83 for higher-voltage processes). Electrochemically, the modified MWCNTs delivered a reversible capacity of 856 mAh g^−1^ after 100 cycles at 0.1 A g^−1^, retaining 88% of their capacity over 500 cycles at 0.5 A g^−1^; at 2 A g^−1^, they maintained 200 mAh g^−1^, outperforming pristine MWCNTs in both capacity and rate performance. Li-ion diffusion coefficients from GITT were an order of magnitude higher than those of untreated CNTs, and Coulombic efficiency stabilized above 98% after initial SEI formation. Although CNT surface functionalization has been widely studied since the advent of MWCNTs, this work is unique in using a one-step acid treatment that simultaneously introduced oxygen-containing functional groups, opened the inner hollow cores, increased carbon disorder, and generated structural defects and voids/nanopores, collectively enhancing Li^+^ storage capacity and accelerating Li^+^ diffusion.

A prelithiated composite anode combining carbon nanotubes (MWCNTs) with Si/graphite (MWCNTs–Si/Gr) was created to boost the initial Coulombic efficiency and long-term stability of lithium-ion full cells. This approach reduces the irreversible lithium loss common in Si and CNT-rich systems [49]. The authors embedded Si alloy particles (0.9–24 μm) within a 10 wt% MWCNT matrix, which formed a fibrous conductive network connecting particles, enhancing conductivity, adhesion to the current collector, and mechanical flexibility under stress. The electrode was prelithiated in vitro using lithium metal in a half-cell format (about 20% lithium reservoir), then recovered, rinsed, and assembled with NMC622 cathodes into full cells. Electrochemically, the prelithiated MWCNTs–Si/Gr full cell reached a first-cycle ICE of 87.2%, compared to 69.5% for the pristine; it reduced the first-cycle irreversible capacity from 62.4 to 25.5 mAh g^−1^, and maintained 129.3 mAh g^−1^ after 100 cycles, outperforming a control with Super P, which achieved only 114.2 mAh g^−1^. Analyses (SEM, Raman, ATR-FTIR, XPS) indicated that electrodes with MWCNTs formed a thinner, Li_2_CO_3_-rich, mechanically robust SEI. The study highlights that CNTs enhance electron conduction and mechanical strength, and prelithiation stabilizes the interphase, facilitating scalable integration into high-energy-density lithium-ion batteries.

A hierarchical MWCNT–Si@Ag@CN composite was synthesized using recycled photovoltaic kerf silicon as the core, with Ag nanoparticles forming conductive bridges and carbon nitride (CN) acting as a multifunctional shell [50]. The authors tackled Si volume expansion and low conductivity by embedding ~100 nm Si within MWCNTs for conductivity, depositing 10–20 nm Ag nanoparticles between Si and CNTs to enhance contact, and coating with graphitic CN to stabilize the SEI and buffer strain. Ag nanoparticles were added via chemical reduction with AgNO_3_ and NaBH_4_, while the CN shell resulted from pyrolysis of dicyandiamide at 550 °C, creating a core–shell–tube structure. Electrochemical tests showed the MWCNT–Si@Ag@CN electrode had a reversible capacity of 1233.3 mAh g^−1^ after 100 cycles at 0.2 A g^−1^, much higher than 478.7 mAh g^−1^ for pure Si, and retained 604.3 mAh g^−1^ at 1 A g^−1^, indicating good rate performance. EIS measurements showed lower charge-transfer resistance and a higher Li^+^ diffusion coefficient (D_Li ≈ 10^−13^ cm^2^ s^−1^) than controls without Ag or CN. This work highlights upcycling silicon waste from photovoltaics: Si nanoparticles for LIB anodes were made by ball milling and acid leaching. Additionally, integrating Ag nanoparticles and a dopamine-derived carbon shell on Si created hierarchical conductive interphases, reducing electrode–electrolyte resistance and greatly improving electrochemical performance.

While incorporating MWCNTs into Si-based anodes has shown promising potential in reducing volume expansion and improving electrical conductivity, several obstacles must be overcome before they can be widely adopted commercially. Structurally, the 1D form of MWCNTs provides efficient electron pathways and mechanical buffering; however, issues such as their tendency to agglomerate, poor dispersion in aqueous or organic slurries, and limited interfacial bonding with Si often lead to uneven network formation and underutilization of active materials [51,52,53]. Additionally, the low tap density and high cost of CNTs restrict their use in high-energy-density electrodes at the industrial level. From a processing perspective, techniques such as in situ CNT growth, sputtering, and freeze-drying offer excellent performance but may lack scalability or compatibility with roll-to-roll manufacturing. Future research should focus on creating hierarchical hybrid networks (e.g., combining CNTs with graphene, amorphous carbon, or polymer binders), surface functionalization to boost interfacial adhesion, and prelithiation strategies to compensate for initial lithium loss typically associated with high-surface-area CNTs. Furthermore, gaining a deeper understanding of CNT–Si interfacial dynamics during long-term cycling—especially under high areal loading and limited electrolyte conditions—will be essential for translating laboratory results into practical battery systems.

**Figure 4:**
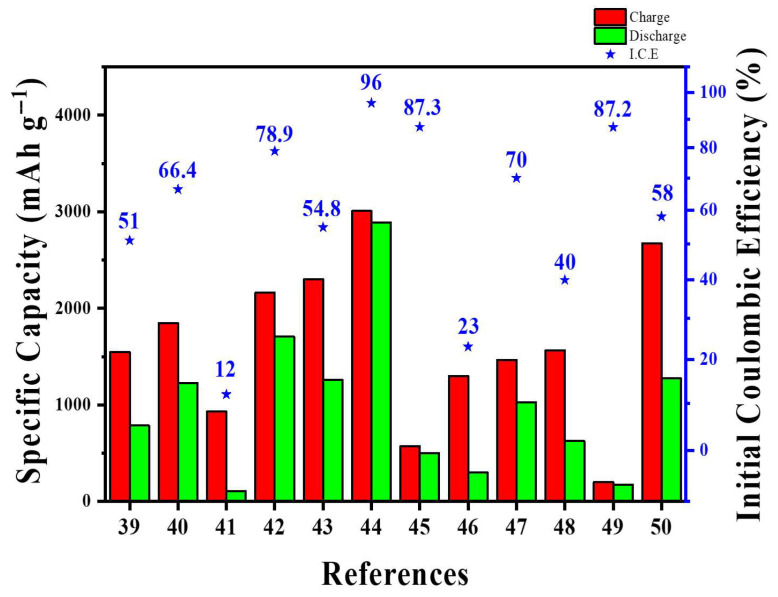
Direct quantitative comparison of various Si-MWCNT architectures in terms of key battery performance metrics.

From a scalability viewpoint, MWCNT–Si architectures show clear trade-offs between vacuum-based and slurry-compatible methods. For instance, sputtered Si on MWCNT buckypaper or tissues achieved excellent interfacial contact but depended on low-throughput vacuum systems and produced limited areal loadings (~1.8–2.0 mg cm^−2^), making it hard to scale beyond pilot production [41,46]. In contrast, spray-dried or freeze-dried Si/MWCNT microspheres and graphite-blended MWCNT-implanted Si composites were produced via atomization and standard slurry mixing, which were directly compatible with current electrode manufacturing systems. Nevertheless, these approaches use more CNTs and demand accurate dispersion control to avoid rapid increase in viscosity, filter clogging, and CNT agglomeration [40,45,47]. Moreover, life-cycle and exergy analyses showed that CVD-based MWCNT production had energy consumption around 10^2^ MJ kg^−1^, so practical industrial designs should minimize MWCNT content while maintaining percolation and tap density [21]. Taken together, the most feasible MWCNT approaches are those that balance CNT loading, electrode density, and roll-to-roll manufacturing compatibility. Table 2 and Figure 4 provide the summary and quantitative comparison of various Si-MWCNT architectures in terms of key battery performance metrics.

### 2.2. SWCNT Strategies for Structurally Stable Silicon Anodes

This chapter examines how SWCNTs mitigate Si-anode failure modes—such as large volume expansion, electrical contact loss, non-uniform lithiation, and unstable SEI—by integrating ultra-lean, long, and flexible 1D networks with particle-level surface engineering. It organizes key design parameters—length/diameter, loading, defect/doping/functionalization, alignment/crosslinking, and processing methods (dry assembly, aqueous slurry with electrostatic assembly, field-assisted arrangement, chemical grafting)—to propose guidelines for SWCNT-enabled, site-specific conductive networks that optimize energy density, durability, and processability under realistic operating conditions.

A binder-free SWCNT film was designed as a long-range conductive network for Si-based anodes. It was directly compared with MWCNT networks, with contact mechanics examined via in situ Raman and kinetic analyses [54]. To restrict Si volume expansion, the anode uses SiO_x_@C secondary particles combined with a highly flexible, high-aspect-ratio SWCNT network whose strong van der Waals interactions keep close contact under large stresses—an advantage over stiffer MWCNTs, which more easily lose contact. The electrode is made using a dry, solvent- and binder-free process: an SWCNT network is assembled under an electric field and integrated with SiO_x_@C on graphite paper, while the control employs a conventional slurry (85% SiO_x_@C/5% MWCNT/10% binder) on Cu. Electrochemically, SiO_x_@C|SWCNT delivers an initial discharge capacity of 1785 mAh g^−1^ with 81.52% ICE, retains 915.9 mAh g^−1^ after 200 cycles at 1 A g^−1^ (658.8 mAh g^−1^ after 500 cycles), maintains roughly 311 mAh g^−1^ at 5 A g^−1^, and shows significantly improved charge-transfer kinetics (Rct 17.85 Ω vs. 80.69 Ω for MWCNT; DLi^+^ increases by about 4 orders of magnitude after 3 cycles). In situ Raman demonstrates that the SWCNT network keeps electrical contact under tensile stresses up to around 6.2 GPa, whereas MWCNTs experience alternating compressive (up to around 8.9 GPa) and tensile (~2.5–2.7 GPa) stresses that correspond with contact loss; the authors attribute the superior kinetics to SWCNT flexibility and stronger intertube/particle van der Waals forces and suggest CNT G-band stress tracking as a general method to monitor contact integrity in Si anodes.

A systematic comparison of CNF and CNT as high–aspect-ratio conductive additives (1–10 wt%) for nano-Si anodes showed that partially replacing Super P with CNF/CNT increases capacity retention from approximately 44% to around 60% and improves fast charge/discharge performance [55]. To mitigate Si’s volume-change effects, the study utilized 1 D percolation networks, CNTs (about ≈ 6–13 nm by 2.5–20 μm) and longer CNFs (roughly ≈ 100 nm by 20–200 μm), which provided line-to-line contacts (compared to point contacts of carbon black), enhanced mechanical integrity, and could reduce electrolyte-channel tortuosity. Additives were introduced via an aqueous slurry method: 30–50 nm Si, LiPAA binder, and Super P (control 6:2:2 by weight), with 1–10 wt% of Super P replaced by CNT or CNF; the slurry was roller-milled and mixed, blade-coated on Cu, calendared, and vacuum-dried before half-cell assembly (test matrix: Baseline, 1/5/10% CNF, 1/5/10% CNT). Electrochemically, CNF or CNT improved 45-cycle retention to approximately 60% (versus around 40–44% baseline), increased fast-discharge capacity to roughly 1650 mAh g^−1^ at 7 C for 10% CNF/CNT, and delivered superior fast-charge performance (1–3 C), with the order being 10% CNF > 5% CNF > 10% CNT. DRT analysis revealed contact-impedance growth from 1.19 to 28.30 Ω·mg (baseline), but only about 2.7 Ω·mg (5% CNF) or 3.1 Ω·mg (5% CNT) at cycle 45. Importantly, morphology studies indicated that >1 wt% CNT could impair coating uniformity (due to CNT agglomeration exposing Cu). In contrast, CNF reduced surface cracking at 1–5 wt%, but cracks grew at 10 wt%, supporting the authors’ recommendation for precise composition control. Higher CNT loadings (5–10 wt%) also degraded coating quality in their process. This study provides practical guidance for battery optimization by systematically replacing carbon black with CNTs and CNFs as conductive additives and quantifying the impact on electrochemical performance. Moreover, mapping performance and cracking versus additive loading emphasizes the importance of optimizing composition; further research on different slurry and coating processes for each additive could enhance its applicability.

A dispersant-free colloidal and interfacial engineering approach was reported, where less-defective graphene oxide (C-GO) and highly oxidized SWCNTs encapsulate micron-scale Fe–Si alloy (SiA) particles to form SiA/nanocarbon (SiA/NC) hybrids. This system requires no additional conductive additives and achieves a high initial capacity (1224 mAh g^−1^, 0.1 C) with 82.3% retention after 100 cycles, as well as a full-cell energy density of 350 Wh kg^−1^ with 65% retention over 200 cycles [56]. To limit Si expansion, each SiA secondary particle was “caged” by a highly crystalline rGO shell and interpenetrated by lithiophilic N-doped SWCNTs, resulting in approximately 8 wt% total nanocarbon, a BET increase from 17 to 58 m^2^ g^−1^, and internal free volume to buffer dimensional changes. The coating was created through kneaded chlorate-based oxidation to produce dispersant-free C-GO/C-SWCNT inks, hydrogen-bonded spray drying of an aqueous SiA/C-GO/C-SWCNT mixture (roughly 88.5:6.5:5 by mass), and chemical reduction with hydrazine (4 mM, 150 °C, 24 h). Electrodes were then fabricated with a composition of 95:0:5 (SiA/NC: binder) without carbon black. Electrochemically, SiA/NC half-cells maintained stable Coulombic efficiency and showed 72.3% capacity retention after 150 cycles, compared to 7.2% for SiA + CB, and exhibited faster Li^+^ transport (GITT DLi^+^ approximately 8.5 × 10^−11^ vs. 5.6 × 10^−11^ cm^2^ s^−1^). In full cells with NCM811, the system reached approximately 350 Wh kg^−1^, with long-term cycling retaining about 65% capacity at 200 cycles. The novelty of this work lies in coupling less-defective C-GO with solution-reduced, N-doped SWCNTs to confine micron-scale Si–Fe alloy particles within a percolating, lithiophilic framework. This architecture eliminates the need for external conductive additives and maintains robust full-cell performance.

Park et al. visualized how SWCNTs preserve nanoscale electron-conduction pathways in SiO/graphite anodes, enabling uniform lithiation and delithiation and thereby reducing capacity fade [57]. To prevent Si-derived pulverization and heterogeneity in volume change, the work used a flexible, high-aspect-ratio 1D SWCNT network that threaded through SiO and graphite regions, equalizing surface potential across particles and reducing microcrack-driven stress concentrations compared to carbon-black-only controls. The SWCNTs were added as conductive additives in conventional SiO/graphite composite electrodes (prepared with and without SWCNTs), and the authors examined the nanoscale electrochemical behavior before and after 90 cycles using Kelvin probe force microscopy (surface-potential mapping), complemented by C-AFM and nanoindentation to identify phases and contact integrity. Electrochemically, the SWCNT system showed over 90% capacity retention after 90 cycles (compared to about 50–74% in the reference repeats), better rate capability, a larger charge–discharge surface-potential gap (indicating more active Li insertion and extraction), and tighter intraparticle potential dispersion (σ ≈ 0.10 V vs. 0.16 V), all consistent with a denser, more reproducible percolation network. The authors emphasize that early-cycle disconnection of electron pathways in SWCNT-free electrodes predicted long-term variability and degradation, whereas SWCNTs maintained uniform reaction sites—offering a mechanistic design criterion for selecting conductive additives in alloy or intercalation composites anodes.

A stress-relieving, electrically conductive capping layer made of carboxylated polythiophene (PPBT) and SWCNTs on Si microparticles (Si MPs) was developed, linking interfacial stress relaxation—measured by in situ Raman—to long-lasting Si-anode performance [58]. To reduce Si volume expansion, 1–5 µm Si MPs were conformally coated with an amorphous PPBT shell that debundled and lithiophilically anchored SWCNTs into a fibrous 3D network. The coating was created using an aqueous, dispersant-free process: poly(diallydimethylammonium chloride, PDDA) pretreatment made Si surfaces cationic, then a PPBT/SWCNT dispersion (formed through π–π interactions) was drop-added for electrostatic bonding, followed by filtering and drying before standard water-based slurry casting. Electrochemically, PPBT/SWCNT@Si MPs achieved 85% ICE at 0.5 A g^−1^, sustained 1715 mAh g^−1^ at 8 A g^−1^, and retained 1894 mAh g^−1^ after 300 cycles at 2 A g^−1^ with about 0.027% decay per cycle; EIS/XPS data show a thinner, LiF-richer SEI, and reduced RSEI/Rct. Additionally, an NCM523 full cell delivered approximately 150 mAh g^−1^ with around 80% retention over 50 cycles. This study is significant because it demonstrates a polymer-anchored SWCNT capping layer that provides mixed ionic/electronic conduction, mechanically locks the nanotube network to Si MPs, and effectively relieves interfacial stress—thus enhancing ICE and high-rate stability.

**Figure 5:**
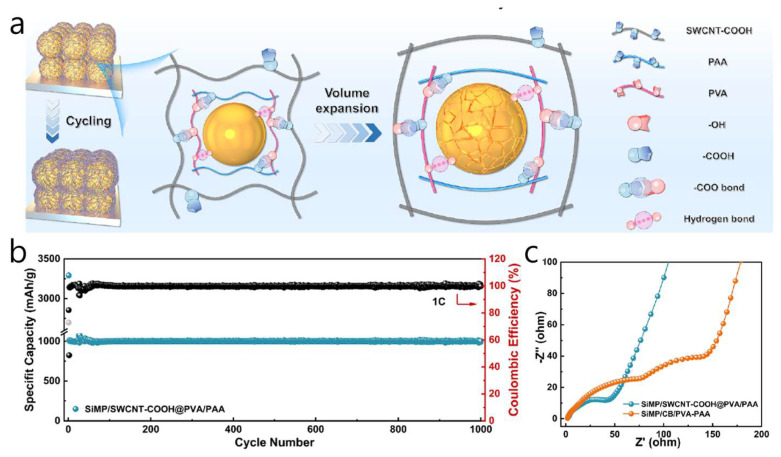
(**a**) Schematic illustration of the 3D conductive skeleton based on coordinated action of PVA-PAA and SWCNT-COOH to alleviate the volume expansion of SiMP anode during charge and discharge processes. (**b**) Constant capacity charge and discharge performance of SiMP/SWCNT-COOH@PVA/PAA tested at 1 C. The black circle represents Coulombic Efficiency. (**c**) Nyquist plots of SiMP/SWCNT-COOH@PVA/PAA and SiMP/CB/PVA-PAA (Reproduced with permission from [59], copyright 2024, Elsevier).

A versatile, self-supporting SiMP/SWCNT-COOH @ PVA-PAA anode was developed, where carboxylated SWCNTs create a highly conductive framework, and a PVA-PAA cross-linked interface improves contacts—resulting in an electrode with ultra-high conductivity and strong mechanics for durable cycling (Figure 5a) [59]. To limit Si microparticle expansion, the SWCNT-COOH network evenly interpenetrates and surrounds SiMPs, while the tough PVA-PAA matrix (benefiting from carboxyl–hydroxyl interactions) reinforces the 3D structure and maintains stability under strain. The coating/electrode is made through a simple solution-sonication process: PAA and PVA are dissolved in DMSO, SiMPs and SWCNT-COOH are ultrasonically dispersed, the mixture is cast into a PTFE mold, dried at 80 °C, then vacuum-dried at 120 °C to form a self-supporting membrane. Electrochemically, the anode reaches approximately 2868.5 mAh g^−1^ at 0.2 C with 96.49% retention after 100 cycles, retains 1795.6 mAh g^−1^ after 300 cycles at 0.3 C (1465.6 mAh g^−1^ at 0.5 C), exhibits rate capacities of 3580/3047/2332/1462 mAh g^−1^ at 0.1/0.2/0.3/1 C, and even maintains about 1000 mAh g^−1^ for 1000 cycles at 1 C (Figure 5b). The SiMP/SWCNT-COOH @ PVA-PAA electrode also displays a significantly smaller high-frequency semicircle, indicating lower charge-transfer resistance and better rate performance. A flexible LFP//SiMP full cell delivers close to 49.5 mAh g^−1^ after 100 cycles. The membrane shows less than 4% resistance change after 5000 bending cycles and has a tensile strength of 46.95 MPa, demonstrating the synergistic “polymer-anchored SWCNT skeleton” concept for flexible, durable Si anodes. The combination of mechanical toughness and flexibility in this hybrid design suggests broad potential for next-generation battery formats, including flexible/wearable Li-ion devices, high-loading pouch/prismatic cells, and emerging solid-state systems. Additionally, for the parameters used in this study (a loading of 1.5–2.0 mg cm^−2^ with an active-material fraction of 0.60), the areal capacity ranges from 3.78 to 5.5–4.30 mAh cm^−2^, based on Si’ s theoretical specific capacity (Qtheo) of 4, 200 mAh g^−1^, or 3.22 to 4.4–4.30 mAh cm^−2^ for Qtheo of 3579 mAh g^−1^. Ultimately, this work is significant because the excellent electrochemical performance (~1000 mAh g^−1^ for 1000 cycles) was achieved at practical areal loadings (≥3 mAh cm^−2^).

**Figure 6:**
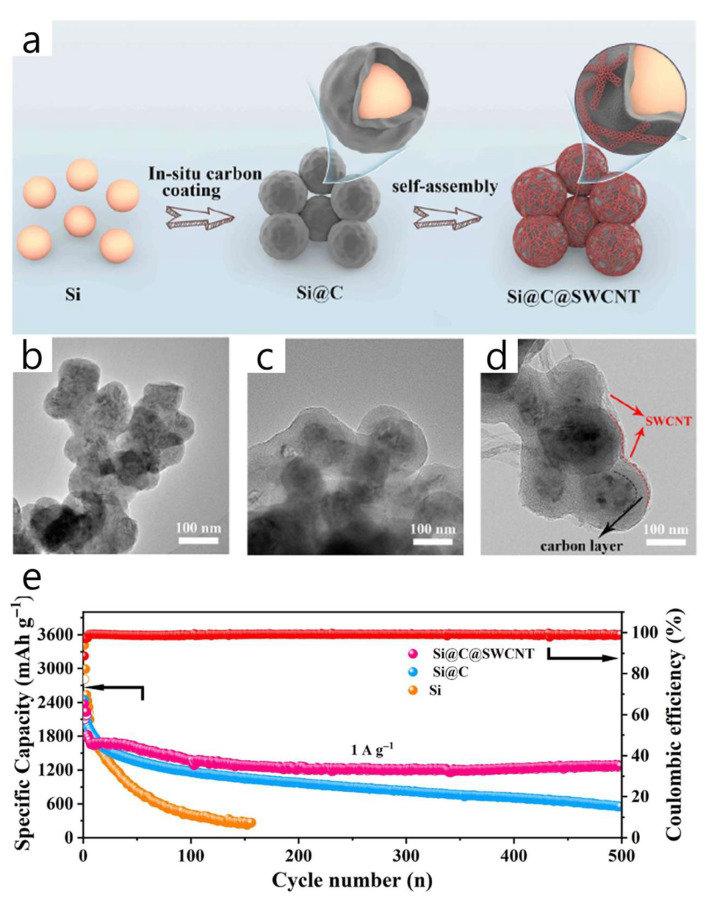
(**a**) Schematic overview of the fabrication process of the Si@C@SWCNT composite. TEM images of (**b**) Si, (**c**) Si@C and (**d**) Si@C@SWCNT composites. (**e**) Long-term cycling stability at 1.0 A g^−1^ for Si, Si@C, and Si@C@SWCNT electrodes. The red circle represents Coulombic Efficiency. (Reproduced with permission from [60], copyright 2023, ELSEVIER).

Kang et al. proposed a dual-layer “Si@C@SWCNT” architecture where a thin, well-anchored SWCNT web is electrostatically attached to a polydopamine-derived carbon shell around commercial Si nanoparticles. This design creates a robust long-range electron pathway and significantly improves cycling durability [60] (Figure 6). To reduce Si volume expansion, the inner N-doped porous carbon shell boosts conductivity and confines Si, while the outer flexible SWCNT mesh (≈3.4 wt%) forms a percolated network that buffers strain, increases surface area (BET 178 m^2^ g^−1^ vs. 17 for Si), and minimizes electrode thickening and cracking after cycling (42.9% thickness increase and ~3 μm cracks vs. 78.6%/7 μm for Si@C and 113.3%/12 μm for bare Si). The coating process involves aqueous PDA coating and carbonization at 750 °C to produce Si@C, cationic surface modification with PDDA, electrostatic self-assembly of oxidized SWCNTs prepared via a modified Hummers’ route, and a mild annealing at 350 °C. Electrodes are then fabricated using conventional aqueous slurry casting. Electrochemically, Si@C@SWCNT delivers 2337 mAh g^−1^ with 88.7% ICE, retains 1267.3 mAh g^−1^ after 500 cycles at 1 A g^−1^ (CE > 98%), and maintains approximately 850 mAh g^−1^ at 8 A g^−1^, indicating faster Li^+^ diffusion (GITT) and reduced charge-transfer resistance (EIS) compared to Si and Si@C controls. Notably, the authors highlight that anchoring a thin SWCNT web directly onto the carbon shell suppresses SEI growth, maintains a 3D conductive network, and outperforms single carbon coatings—providing a practical “outer-SWCNT/inner-carbon” design rule for high-rate, long-life Si anodes. However, the paper reports an active-mass loading of 0.8–1.0 mg cm^−2^ and a Si fraction of 0.691; the corresponding theoretical areal capacity is 2.32–2.90 mAh cm^−2^ (Qtheo = 4200 mAh g^−1^) or 1.98–2.47 mAh cm^−2^ (Qtheo = 3579 mAh g^−1^), which approaches but remains just below the ≥3 mAh cm^−2^ criterion for practical areal loading within the stated range.

Recently, some studies have systematically analyzed how the type and morphology of CNTs influence the electrochemical performance of Si-based LIB anodes. Because these findings provide critical guidance for CNT selection and anode design in manufacturing, we include them in this review. A study revealed that SWNTs exploit lithiation-induced expansion in Si/SiO_x_ to trigger strain-driven interfacial reactions, forming Li–C (sp^3^) and Si–C bonds, which stabilize pulverized Si while reconstructing a mechanically reinforced, ion- and electron-conductive 3D network [61]. To moderate Si volume expansion under practical loading, the study used an ultra-low 1 wt% of flexible SWNTs that experienced about 14–16.5% tensile strain during lithiation (operando Raman), enabling a percolated, compliant scaffold that accommodated expansion without sacrificing tap density. SWNTs were grown by catalytic gas-phase CVD in a quartz-tube reactor at 1200 °C using ferrocene (1.0 wt%) and thiophene (0.5 wt%) as catalyst/promoter, purified in HCl, dispersed with CMC, then either vacuum-filtered with SiO_x_ to produce binder-free films for mechanistic tests or incorporated at 1 wt% into slurry-coated graphite/SiO_x_@C composite anodes for device-level evaluation. Electrochemically, the SWNT-enabled graphite–SiO_x_@C anode delivered 600 mAh g^−1^ at 0. 05 A g^−1^ with ICE ≥ 81. 9%, exhibited only approximately 0.035% capacity decay per cycle at 0.05 A g^−1^ (CE 99.9% after 20 cycles), and in NCM 811 full cells attained 474 mAh g^−1^ at 0. 5 A g^−1^ with 81. 7% retention over 400 cycles and ≈ about 493 Wh kg^−1^ energy density; rate capability reached 423 mAh g^−1^ at 2.5 A g^−1^. The key novelty of the paper is that, unlike rigid MWNTs, strained SWNTs (>14% strain) promote irreversible Li–C/Si–C bond formation and sharply reduce the charge-transfer resistance, with Rct dropping from 214.6 Ω to 12.4 Ω within the first three cycles, thereby establishing a chemomechanical coupling mechanism that justifies the use of site-specific, ultra-lean SWNT additives for practical Si–graphite anodes.

Another study reported the “acupuncture effect” in Si-based anodes, showing that compressive stress on CNTs generated by Si/SiO_x_ volume expansion can puncture the SEI and carbon coating, producing a LiF-rich, high–Li^+^-barrier interphase. He et al. correlated CNT-induced stress with SEI diffusion barriers and identified long, slender CNTs as optimal for durable cycling [62]. To prevent expansion-driven interfacial failure, the study contrasted flexible high–aspect ratio SWCNTs/MWCNTs (approximately 1–1.044 mm in length; slender diameter) against short, thicker CNTs (around 2–2.5 µm), discovering that long CNT networks wrapped particles and maintained contact, while short CNTs imposed GPa-level compressive stress that pierced the SEI and facilitated LiF accumulation. Four SiO_x_@C electrodes were fabricated with long-SWCNT, short-SWCNT, long-MWCNT, and short-MWCNT networks and analyzed through temperature-dependent EIS (Arrhenius analysis of SEI Li^+^ diffusion), in situ Raman (CNT G-band stress), TOF-SIMS/XPS (SEI composition), and cryo-TEM. These analyses established a linear relationship between maximum CNT compressive stress and SEI energy barrier. Electrochemically, long-SWCNTs delivered the highest 200-cycle capacity (~989 mAh g^−1^), followed by long-MWCNT (~883 mAh g^−1^), short-SWCNT (~786 mAh g^−1^), and short-MWCNT (~712 mAh g^−1^). Initial discharge capacity and ICE were slightly higher for long versus short CNTs. The extracted activation energies for Li^+^ transport through the SEI ranked as SW-long (27.62 kJ mol^−1^) < MW-long (34.34) < SW-short (44.84) < MW-short (57.48). A new finding of this paper is that when MWCNTs are sufficiently long and thin, they behave similarly to SWCNTs: their one-dimensional percolation reduces compressive stress, strengthens electrical contact, and promotes a more resilient, diffusion-stabilized SEI, collectively enhancing the performance of Si-based anodes.

Kim et al. also systematically uncovered how adding an ultra-lean amount of CNTs to densely packed Si–graphite composite (SGC/G) anodes in industrial-scale pouch cells suppressed electrode swelling and stabilized long-term cycling by maintaining electron pathways and reducing strain-induced interfacial reactions [63]. To prevent Si volume expansion, the study designed a one-dimensional, high–aspect-ratio CNT network that increased true contact area with the Si-coated graphite, improved inter-particle adhesion (≈159 N m^−1^ vs. 113 N m^−1^ for CB), and distributed stress—resulting in significantly less cracking and much lower irreversible expansion during cycling. The electrodes were made using practical aqueous slurry processing: SGC produced by CVD of Si (SiH_4_, 475 °C) on graphite, then pitch-carbon coated and annealed, blended with graphite (≈24.5:75.5), and cast with 0.1 wt% CNT (or 1 wt% CB) and CMC/SBR binder to a density of 1.5 g/cm^3^; full cells paired the anode with Ni-rich single-crystal NCM (N/P ≈ 1.1). Electrochemically, CNTs reduced first-charge swelling to about 27% (compared to approximately 170% with CB), decreased irreversible thickening by roughly six times over 50 cycles, and in pouch cells delivered about 94.6% capacity retention at 25 °C over 100 cycles. They also enabled faster rate and fast-charge response (+13% at 3 C; roughly 8% better retention under fast-charge protocols), improved low-temperature cycling (close to 100% over 30 cycles at −10 °C), and lowered resistance as measured by HPPC/EIS (e.g., 59.1 mΩ vs. 66.8 mΩ at 50% DOD; 0.033 Ω vs. 0.042 Ω after cycling). Notably, in situ dilatometry, SEM-BSE, and TEM-EDS analyses led the authors to suggest a “binding-and-bridging” mechanism—long, slender CNTs homogenized lithiation, slowed crack propagation, and limited SEI growth at both the particle and electrode levels, offering a clear CNT-selection and usage guideline (ultra-low loading, high aspect ratio) for commercial Si-graphite anodes. The main scientific contribution of this paper is in elucidating, across both lab-scale electrodes and practical pouch full cells, how SWCNTs suppressed Si expansion and enhanced interfacial adhesion between inter-particle contact/slurry coating layer and the current collector.

In conclusion, SWCNT-enabled Si anodes have advanced from proof-of-concept to mechanism-aware engineering, but several challenges remain before widespread adoption. At practical areal loadings and high electrode densities, designers need to balance ultra-lean percolation (to maintain energy density) against contact durability; binder-free films and high SWCNT contents can complicate calendaring and wetting, while insufficient SWCNT amounts risk network failure under stress. Variability in processing (dispersants, aqueous compatibility, catalyst residues) further underscores the need for reproducible, surfactant-minimal methods [64,65,66,67,68]. These efforts aim to transform SWCNT networks from laboratory concepts into reliable, manufacturable solutions for managing Si anode expansion.

**Figure 7:**
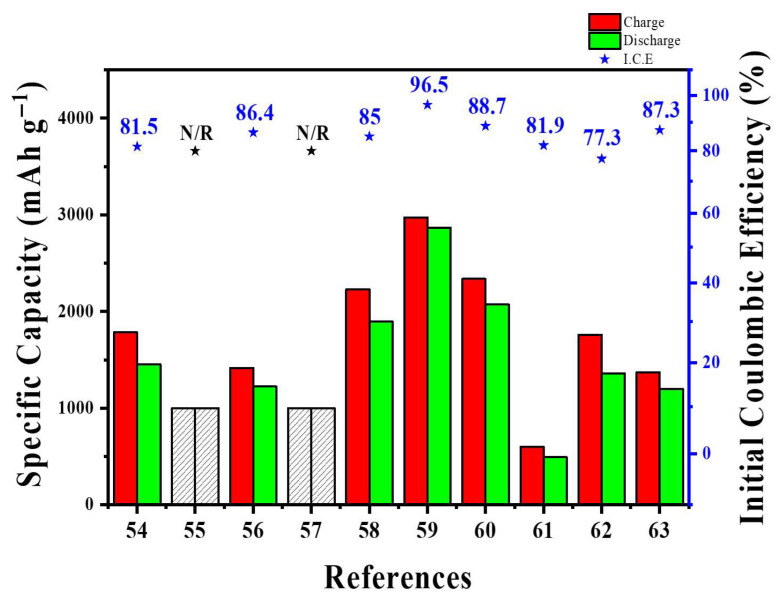
Direct quantitative comparison of various Si-SWCNT architectures in terms of key battery performance metrics.

At the pilot level, SWCNT–Si architectures demonstrate a similar trade-off between network quality and process flexibility. Self-standing SiMP/SWCNT–COOH@PVA–PAA membranes and PPBT/SWCNT@Si microparticles exhibited excellent long-term cycling and full-cell stability; however, they rely on multi-step infiltration, vacuum filtration, or polymer encapsulation methods that are challenging to scale up in high-speed coating lines [58,59]. Conversely, “ultra-lean” SWCNT additions (~1 wt%) to SiO_x_@C or Si/graphite blends can be directly dispersed into conventional slurries and have already been tested in NCM811 or SC-NCM pouch cells at practical areal loadings [61,63]. Still, even at these low levels, the high aspect ratio of SWCNTs increases slurry viscosity, requiring optimized dispersant systems, mixing procedures, and careful control of metal-catalyst residues from SWCNT production [54,55,56]. These examples suggest that scalable SWCNT-based designs will require low CNT fractions, slurry-processed architectures rather than free-standing papers, and rheology-based analyses of slurry flow. Table 3 and Figure 7 provide the summary and quantitative comparison of various Si-SWCNT architectures in terms of key battery performance metrics.

### 2.3. Graphene Strategies for Structurally Stable Silicon Anodes

Graphene, owing to its outstanding electronic conductivity, mechanical flexibility, and chemically tunable surface, has emerged as a versatile platform for managing the significant volume change in Si anodes [69,70,71,72,73]. This chapter reviews graphene-based strategies that stabilize Si expansion across various length scales. Recently, graphene has been seen not just as a passive coating but as an active, dynamic interface and structural regulator that provides high areal and volumetric capacities and ensures durability in full cell performance.

Son et al. reported an early, pioneering study on a Si-carbide-free, multilayer-graphene coating grown directly on Si nanoparticles that enabled record volumetric performance in Li-ion anodes and full cells [74]. The coating consisted of 2–10 well-aligned graphene layers conformally anchored to Si; its 2D, layered architecture formed a high-percolation conductive network (12.8 S cm^−1^ at just 1 wt% C; 38.3 S cm^−1^ at 5 wt%) and accommodated Si expansion via interlayer sliding, eliminating the need for pre-built voids. To achieve this, the authors developed a CO_2_-assisted CH_4_ CVD method that activated a thin SiO_x_ surface to catalyze graphene growth and suppressed SiC formation. Electrochemically, Gr–Si half-cells delivered approximately 2500 mAh cm^−3^ with strong cycling and rate stability, while 18650-type full cells with LiCoO_2_ achieved around 972 Wh L^−1^ initially and about 700 Wh L^−1^ after 200 cycles (roughly 1.8× and 1.5× better than graphite controls), with up to ~85% capacity retention over 200 cycles depending on graphene loading. In situ TEM further revealed that intact shells allowed fast, uniform Li transport and a mechanical “clamping” effect that enabled approximately 30% diameter (around 220% volume) expansion without fracture, whereas defects led to rupture. Academically, this paper advanced the field by proposing a CVD-grown graphene coating on Si surfaces, a long-standing and extensively studied approach. Additionally, this work provided key inspiration for subsequent studies on Si–graphene composite anodes.

**Figure 8:**
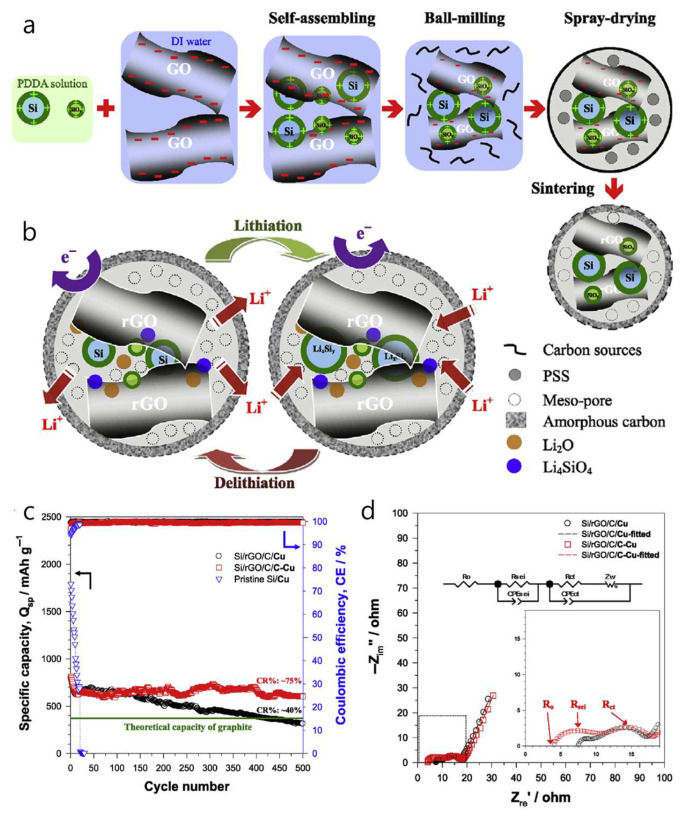
(**a**) Synthesis procedure of the ternary hierarchical Si/rGO/C composite microsphere. (**b**) Schematic diagram of electron (e^−^), Li-ion (Li^+^) pathways, and synchronous formation of inert products such as Li_2_O and Li_4_SiO_4_. (**c**) Cycling performance of the Si/rGO/C composite electrodes using bare Cu and CeCu foils as the current collector at 400 mA g^−1^ in comparison with the pristine Si/Cu electrode. (**d**) Electrochemical impedance spectra with the corresponding equivalent circuit model (inset) of the Si/rGO/C composite coated on bare Cu and CeCu foils after Cerate test. (Reproduced with permission from [75], copyright 2023, Elsevier).

Ternary hierarchical Si/reduced-graphene-oxide/carbon (Si/rGO/C) composite microsphere was designed, showing that coupling the anode with a carbon-coated copper current collector (C-Cu) enhances rate performance and long-term stability compared to bare Cu (Figure 8a) [75]. The Si volume expansion is buffered by a dual-carbon matrix, where rGO sheets and a ~1–2 nm amorphous carbon layer encapsulate Si + SiO_x_ nanoparticles. Abundant interior mesopores (~17 nm on average) inside ~0.7–5 µm spheres further accommodate expansion, with HRTEM revealing rGO (002, ~0.33 nm) and Si (111, ~0.31 nm) lattice spacings. The coating and assembly process relies on electrostatic self-assembly of PDDA-modified, positively charged Si + SiO_x_ (ζ ≈ +45.7 mV) with negatively charged GO (ζ ≈ −34.6 mV), followed by high-energy ball-milling, PSS-templated spray-drying, and 800 °C Ar/H_2_ reduction to produce the porous Si/rGO/C spheres. According to the authors, when initially discharged, amorphous nano-SiO_x_ reduces to Si and either amorphous Li_2_ O (detectable at 528.3 eV in the O 1 s spectrum via synchrotron-based XPS) or crystalline Li_4_ SiO_4_. Along with LiF, these species form a dense interphase that is ionically conductive but electronically insulating. This layer is crucial for stabilizing the Si anode by accommodating volume changes and preventing continuous electrolyte decomposition (Figure 8b). Electrochemically, the Si/rGO/C on C-Cu delivers an initial lithiation/delithiation capacity of 1643/1005 mAh g^−1^ with about 61.2% initial coulombic efficiency (ICE) at 100 mA g^−1^ and retains around 602 mAh g^−1^ after 500 cycles at 400 mA g^−1^ (CR ≈ 75%, CE ≈ 99.7%), outperforming the same composite on bare Cu (~314 mAh g^−1^) (Figure 8c). Impedance analyses show reduced ohmic and charge-transfer resistances with C-Cu (Figure 8d). The authors attribute these improvements to a synergistic design—structural buffering by rGO/C-confined mesopores combined with a more adhesive, electrolyte-wetted C-Cu interface (contact angle < 5° vs. ~24° on Cu), which lowers R_0_. They also note early capacity loss from FEC consumption during formation. Despite the manufacturing advantages of the electrostatic self-assembly and spray-drying process, and the benefits of the carbon-coated Cu current collector, the relatively low initial coulombic efficiency (~61%) limits near-term applicability, highlighting the need for ICE improvement measures.

A free-standing “sandwich” N-doped graphene@Si@hybrid silicate (N-G@Si@HSi) anode was proposed with a 3D bicontinuous nanoarchitecture that simultaneously addressed Si’s volume change, poor conductivity, and SEI instability. It delivered ultralong cycling (e.g., 817 mAh g^−1^ at 5 C for 10,000 cycles) and demonstrated stable LiFePO_4_ full cells [76]. To buffer expansion, the design used a seamlessly interconnected nanoporous N-doped graphene scaffold as a flexible, conductive backbone; a conformal Si layer (optimized at ~59 nm) as the active host; and an outer amorphous hybrid silicate (SixOy with –OCH_3_/–OCH_2_CH_3_/–CH_2_CH_2_SH) that was mechanically compliant, ion-conductive (~10^−6^ S cm^−1^), and confined SEI formation to the exterior surface. Electrochemically, N-G@Si-30@HSi showed initial Coulombic efficiency boosted to ~81.0% (vs. 74.9% without HSi), with a Coulombic efficiency above 99% after 10 cycles, high-rate capacity decreasing from 1871 to 1036 mAh g^−1^ (0.2→5 C), and over 1286 mAh g^−1^ sustained across more than 1400 cycles at 0.5 C. EIS indicated stable charge-transfer resistance, and long-term tests retained 817/537 mAh g^−1^ after 10,000 cycles at 5/10 C. LiFePO_4_ full cells delivered 129.5 mAh g^−1^ at 0.2 C with approximately 95–96% capacity retention over 100 cycles. This architecture demonstrated state-of-the-art durability under aggressive fast-charge and fast-discharge protocols, retaining around 817 mAh g^−1^ at 5 C and approximately 537 mAh g^−1^ at 10 C over 10,000 cycles—a benchmark result for high-rate Si anodes that effectively suppressed mechanical and interfacial failure modes. This approach is notable for assembling a CVD-grown, graphene-derived 3D bicontinuous framework before incorporating Si particles. The resulting N-G@Si@HSi anode concurrently restrained Si expansion and overcame persistent limitations, including poor electronic conductivity and SEI instability, in both half-cell and full-cell tests.

A pressure-tuned Si@graphene layered composite (p-Si@GN) was developed to enhance the Si/graphene contact interface; the optimized p-Si@GN40 (40 MPa) delivered 2096.9 mAh g^−1^ at 300 mA g^−1^, 706.4 mAh g^−1^ at 3000 mA g^−1^, and 82.6% capacity retention after 150 cycles [77]. The structure featured Si nanoparticles closely wrapped by graphene layers; compression reduced the graphene interlayer spacing (XRD (002)/(004) reappearance), strengthened interfacial contact, built a continuous graphene conductive network, increased electrode density, and maintained flexibility. Synthesis involved hydrothermal assembly of a Si/GO hydrogel, freeze-drying, brief high-pressure pressing (20–60 MPa; optimum at 40 MPa), and 500 °C Ar annealing to produce self-supporting p-Si@GNx electrodes. Electrochemically, p-Si@GN40 showed an initial charge capacity of 2096.9 mAh g^−1^ with elevated ICE (90.7% versus 81.8% for the unpressed control), high-rate capability (706.4 mAh g^−1^ at 3000 mA g^−1^), long-term stability (1730 mAh g^−1^ after 150 cycles; 82.6% retention), high areal capacity (4.68 mAh cm^−2^, 150 cycles), and reduced interfacial resistance by EIS. Unlike other approaches that depend on chemical modifications to strengthen the Si–coating interface, this paper demonstrates that a simple adjustment of compaction pressure can improve the silicon–graphene contact and significantly enhance battery performance, highlighting practical industrial applications.

The development of a self-adaptive graphene-coated Si suboxide/graphite blended anode (SiO_x_@Gr/AG) was reported [78]. In this design, multilayer graphene sheets conformally wrapped SiO_x_ microparticles and acted both as a 2D conductive network and as a solid lubricant within a practical composite electrode (93 wt% active; electrode density ≈ 1.5 g cm^−3^) based on artificial graphite, mitigating local damage while maintaining electronic pathways. The graphene coating was prepared by a simple mix–centrifuge–wash sequence with commercial SiO_x_ (repeated 1–3×) and vacuum drying, before blending with AG for electrode fabrication. Electrochemically, the blended SiO_x_@Gr/AG half-cell recorded about 680 mAh g^−1^ on the first cycle and approximately 620 mAh g^−1^ at 0.5 C, with 71.8% retention over 50 cycles and CE reaching 99.3% by the 9th cycle; rate performance remained around 400 mAh g^−1^ at 2 C, while EIS showed lower RCT than the graphene-free control. In pouch full cells with NCM, the SiO_x_@Gr/AG anode retained approximately 69% capacity over 300 cycles and achieved higher volumetric capacity than AG-only or uncoated SiO_x_/AG. This work provides an analytical assessment of the impact of repeated graphene coating on the electrochemical properties of AG/SiO_x_ composite anodes. Graphene served concurrently as a solid lubricant and as a percolating conductive network between the active components, permitting a reduction in the carbon-black fraction, thus offering the most practical current model (≥3 mAh cm^−2^ of areal capacity) for applying nanocarbon to silicon-based anodes.

Recently, to address the significant volume expansion of Si anodes, hetero-material bilayer-coating strategies that combine nanocarbon with additional layers such as organic polymers, metal oxides, or alternative carbons have been reported. Double-core–shell porous Si@graphene@ solvent-confined-TiO_2_ (p-Si@G@sc-TiO_2_) microspheres were prepared via electrostatic self-assembly and a solvent-confined TiO_2_ monomicelle process, achieving high initial Coulombic efficiency and excellent rate and cycling stability for Li-ion anodes [79]. The design buffers Si swelling with a hierarchical porous Si core composed of interconnected Si slices and hundreds-of-nanometer channels, while an ultrathin graphene inner shell and a conformal anatase TiO_2_ outer shell (≈0.4–0.8 nm graphene; ≈1.8–5.1 nm TiO_2_) stabilize the framework, provide electron and ion pathways, and shield the inner Si from the electrolyte. Electrochemically, p-Si@G@sc-TiO_2_ exhibited 2597.9 mAh g^−1^ at 0.2 A g^−1^ with an 80.59% initial Coulombic efficiency, retained 1005.1 mAh g^−1^ after 300 cycles at 2 A g^−1^ (68% retention), and maintained 802.53 mAh g^−1^ at 8 A g^−1^. It also showed a markedly reduced Rct (to 12.64 Ω after 10 cycles) with higher Li^+^ diffusion coefficients compared to p-Si, p-Si@G, and sol–gel-coated controls. The main advantage of this study is in the decoupling of functions at the Si interfaces: graphene suppresses volume expansion, while a uniform TiO_2_ shell, lithiated into a conductive and mechanically resilient Li_x_TiO_2_ layer, shields Si from the electrolyte, reduces parasitic reactions, and supports stable cycling.

Zhang et al. developed a “functionalization-assisted ball-milling” method to covalently bond Si nanoparticles with graphene (Si@APTES/f-Gr), overcoming issues with poor dispersion and weak interfacial contact to create high-performance Li-ion anodes [80]. The authors reduce Si volume change by using approximately 40 nm Si NPs with –NH_2_ groups that electrostatically or covalently attach to a flexible 3D graphene scaffold. Si was evenly distributed on graphene surfaces or embedded between layers, with FTIR showing COO–NH_3_^+^ linkages consistent with strong interfacial bonds. The synthesis involved a modified Birch reduction to functionalize graphene with carboxyl groups (using liquid NH_3_, Na, 6-bromohexanoic acid), piranha hydroxylation of Si followed by APTES silanization, then self-assembly by ball-milling Si@APTES (25 mg) with f-graphene (100 mg) in DMF for 4 h. Electrochemically, Si@APTES/f-Gr demonstrates an initial discharge capacity of 2919.4 mAh g^−1^ with 66.9% ICE, maintaining 1516.23 mAh g^−1^ after 100 cycles at 100 mA g^−1^, and around 1151.5 mAh g^−1^ over 1000 cycles at 1000 mA g^−1^. It also exhibits strong rate capability up to 5 A g^−1^, with capacity recovering to roughly 1743 mAh g^−1^ when returning to 0.1 A g^−1^. Literature reports FTIR/EIS data indicating COO–NH_3_^+^ bonding and a significantly lower charge-transfer resistance (~117 vs. 546 Ω), along with a higher Li^+^ diffusion coefficient (~6.4 × 10^−11^ cm^2^ s^−1^), supporting the idea that robust interfacial chemistry combined with a scalable ball-milling platform can help bridge the gap toward mass production. However, the dataset presented in the study consisted of thin laboratory electrodes; at a mass loading of 0.1–0.3 mg cm^−2^ and an effective Si fraction of 0.1841, the corresponding areal capacity ranged from 0.077 to 0.232 mAh cm^−2^ (Qtheo = 4200 mAh g^−1^), or 0.066 to 0.198 mAh cm^−2^ (Qtheo = 3579 mAh g^−1^), which remains well below practical levels (≥3 mAh cm^−2^).

Although physical coupling between Si and nanocarbon layers can help reduce swelling and shield the electrolyte–interface, a chemically bonded, tighter interface has been reported to offer better protection for Si. Li et al. described a covalently bonded, highly graphitic carbon shell of stacked graphene layers tightly anchored to microsized Si (Si-co-HGCS) [81]. This architecture dynamically maintained electrical and mechanical contact during significant Si deformation, enabling thick, high-loading anodes with areal capacities up to 5.6 mAh cm^−2^ and volumetric capacities of 2564 mAh cm^−3^. The Si expansion was buffered by interlayer sliding within the graphitic shell, while strong Si–C bonds prevented gap formation at the Si/carbon interface, supporting electron/ion transport and preventing shell fracture. The coating was created using a copper-catalyzed CVD process, resulting in a highly graphitized shell (≈3.8 wt% carbon by TGA). Electrochemically, Si-co-HGCS maintained 747 mAh g^−1^ after 500 cycles at 1 C, reached 1820 mAh cm^−3^ at 0.3 C, and in high-mass-loading electrodes (initial 7.09 mAh cm^−2^), delivered a reversible 5.6 mAh cm^−2^, outperforming physically coated controls. The key feature of this study is the engineering of covalent Si–C interfaces via copper-catalyzed CVD: a more graphitic, sliding graphene shell that dynamically preserved electrical and mechanical contact during Si deformation, while covalent anchoring prevented core–shell gap formation, thus enabling thick, high-loading electrodes and stable cycling.

**Figure 9:**
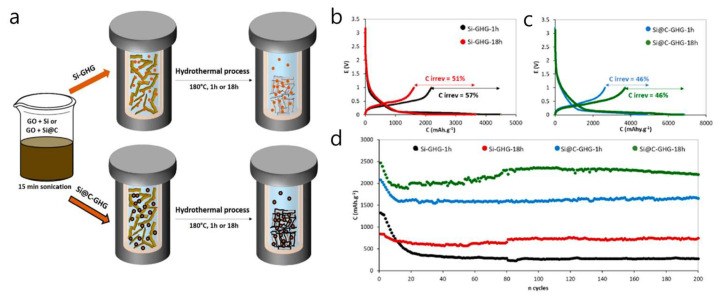
(**a**) Scheme of the synthesis of Si-GHG (top) and Si@C-GHG (bottom). First cycles at C/20 with the corresponding irreversible capacities for Si-GHG samples (**b**) and Si@C-GHG samples (**c**). (**d**) Cycling performances (discharge capacities) of the different samples at C/5 charging/discharging rate [82].

An in situ Si@C–graphene hydrogel (GHG) composite, embedding carbon-coated Si nanoparticles within a 3D graphene network, was developed to stabilize Si anodes [82]. In half-cells without extra conductive additives, the composite delivered a high specific capacity (up to ≈2200 mAh g^−1^) with good stability over 200 cycles and an average coulombic efficiency > 99%. The Si volume/oxidation issue was mitigated by a thin protective carbon shell around the Si nanoparticles, which were homogeneously dispersed throughout the GHG. The Si@C nanoparticles were produced by laser pyrolysis and then assembled into a GHG via a single-step in situ hydrothermal reduction of graphene oxide (1 h or 18 h), forming around the Si@C and resulting in electrodes formulated without added carbon black (Figure 9a). Electrochemically, the Si@C-GHG-18 h electrode reached 2205 mAh g^−1^ after 200 cycles at C/5 with an ≈99% average CE. It shows ≈2500 mAh g^−1^ at C/5 with 55% retention at 2C (recovering ~1900 mAh g^−1^ when the rate returns to C/5), and outperformed both a simple GHG/Si@C physical mixture and a standard Si@C/CMC/CB formulation in cycling stability (Figure 9b–d). This study introduces an in situ hydrothermal process that preserved Si particle performance while reducing graphene oxide, producing a 3D graphene architecture and uniformly distributing Si@C NPs in the GHG matrix. Nevertheless, the high first-cycle irreversible capacity (ICE = 46–57%) remains to be resolved.

Similar to studies on carbon nanotubes, research on the size effects of graphene for silicon surface coatings has been documented. Liang et al. adjusted the lateral size of rGO to create core–shell Si@rGO composites, demonstrating that small-sized rGO sheets (Si@rGO(S)) significantly enhanced cycling stability and rate performance compared to larger rGO sheets (Si@rGO(L)) and pure Si by fostering a stable, LiF-rich SEI and lower interfacial and charge-transfer resistances [83]. The expansion of Si volume was mitigated by an rGO outer shell forming a compact core–shell secondary particle; smaller rGO sheets generated low-tortuosity ion pathways and greater surface area/mesopore volume (~3–5 nm), facilitating multiple Li-ion channels and improved swelling control (+82% versus +117% thickness change after 100 cycles for S versus L). The coating process involved spray drying Si with GO, followed by thermal reduction (950 °C, Ar, 1 h) to produce Si@rGO core–shell agglomerates measuring 3–4 µm. Electrochemically, Si@rGO(S) achieved higher capacity (2586 vs. 2181 mAh g^−1^ at 0.1 C), a high initial CE (~93%), cycling efficiency of ≥99.5% after formation, and 78% capacity retention after 150 cycles (compared to 62% for L and 16% for pure Si). Rate tests up to 3 C revealed superior kinetics, while EIS measurements indicated consistently low RSEI and Rct for Si@rGO(S). Unlike prior research where thin, high–aspect-ratio SWCNTs outperformed thicker CNTs in Si-based batteries, this study illustrates that smaller lateral-size rGO flakes improved cycling stability and rate capability by reducing ion-transport pathways and increasing accessible surface area. Since the graphene used here is chemically reduced graphene oxide with a relatively high defect density, a direct comparison with near-defect-free CNTs is not entirely appropriate. Nonetheless, as rGO is the most commercially used graphene derivative, this work provides a practical benchmark for understanding how rGO’s structural features influence Si-based anodes.

A 3D-printed, freestanding Si/reduced-graphene-oxide (Si/rGO) anode with a porous grid-like structure for flexible pouch cells was designed to leave void spaces for alloying-induced expansion and to outperform conventional Si/rGO film electrodes in both half- and full-cell tests [84]. The Si volume expansion was reduced by using 50 nm Si nanoparticles uniformly coated with flexible, conductive rGO films. The grid features approximately 400 µm holes on a 500 µm pitch, with additional 1–10 µm pores created through freeze-drying, collectively providing short Li^+^ diffusion paths and buffering against pulverization. Fabrication involved extruding a Si/GO ink—made by dispersing Si (50 nm) in GO at a 1:2 mass ratio (≈70 mg mL^−1^)—followed by freeze-drying, thermal reduction at 800 °C for 2 h, and a brief pressing at 10 MPa to produce a freestanding electrode. Electrochemically, the 3D-printed anode delivered 930.64 mAh g^−1^ at C/2 and retained about 68.89% capacity after 350 cycles in half-cells. In NMC532 full cells, it retained 76.08% capacity at C/2 and 64.86% after 100 cycles. Electrochemical impedance spectroscopy (EIS) indicated reduced Warburg diffusion resistance compared to film controls. This work transforms a common limitation of solution-based 3D printing—imperfect surface finish and uncontrolled voids—into a design advantage by creating a porous, grid-like graphene/Si framework. This framework provides short Li^+^ diffusion channels and ensures continuous electronic percolation, which not only enhances ionic and electronic conductivity but also leverages the low-temperature, low-cost, material-efficient nature of 3D printing to develop flexible pouch cells, showing strong potential for future cell manufacturing.

In many earlier studies, graphene was applied by coating Si with highly dispersible graphene oxide, followed by a post-reduction process. However, recent developments introduced a one-step, aqueous quasi-defect-free reduced graphene oxide (QrGO) coating that forms core–shell SiO_x_/C/QrGO composites. These composites enable higher Si loading (20 wt% versus approximately 5 wt% in commercial versions) and achieve full-cell capacities roughly twice those of commercial SiO_x_/C after 500 cycles with NCM 622 [85]. The structure consists of a SiO_x_/C core (roughly 50 nm of amorphous carbon) wrapped by a wrinkled, multilayer QrGO shell (tunable thickness of about 20–80 nm; few-layer sheets less than 1 nm thick). This design improves conductivity, buffers volume expansion, reduces surface roughness (Ra from 3.3 to 1.1), and enhances wettability (contact angle from 109.3° to 73.9°), promoting uniform electrode formation. QrGO was produced via a modified Brodie process combined with HI reduction, then directly spray-dried with commercial SiO_x_/C (10:1 solid ratio, approximately 1 wt% slurry) to form the shell without requiring post-reduction. The QrGO layer displayed strong adhesion and maintained structural integrity even after heating at 600 °C, aided by cation–π interactions. In half-cell tests, SiO_x_/C/QrGO retained around 60% capacity after 50 cycles at 0.5 C (compared to 18–26% for control samples) and showed reduced Rct/RSEI. In electrodes blended with graphite (containing 20 wt% Si), this structure proved effective. Unlike CNTs, graphene’ s synthesis and scale-up remain less standardized, often resulting in lab-produced graphene with variable defect densities and morphologies that influence composite behavior. In this study, a modified Brodie method was used to produce low-defect graphene suitable for Si coating, and process parameters during graphene–Si hybridization (such as thermal and chemical reduction) were carefully controlled, leading to improved reproducibility compared to previous reports.

From a graphene-focused perspective, the main challenge today is consistency: studies seldom report graphene quality metrics (layer number, lateral size distribution, ID/IG and I_2_D/IG ratios, interlayer spacing, O/C ratio, dopant/defect density, conductivity) alongside electrode conditions (density, calendaring pressure, Si fraction, electrolyte/additives, N/P ratio, liquid-to-capacity ratio). This makes it difficult to compare or reproduce structure-performance relationships [86,87,88]. Without prelithiation, the target metrics are ICE > 90%, at least 80% capacity retention after 500 cycles at ≥3 mAh cm^−2^, electrode swelling under 15%, and less than a twofold increase in cell resistance. These benchmarks position graphene as more than a passive coating; instead, it functions as a purpose-built, active interfacial skeleton that enables EV-grade Si anodes.

**Figure 10:**
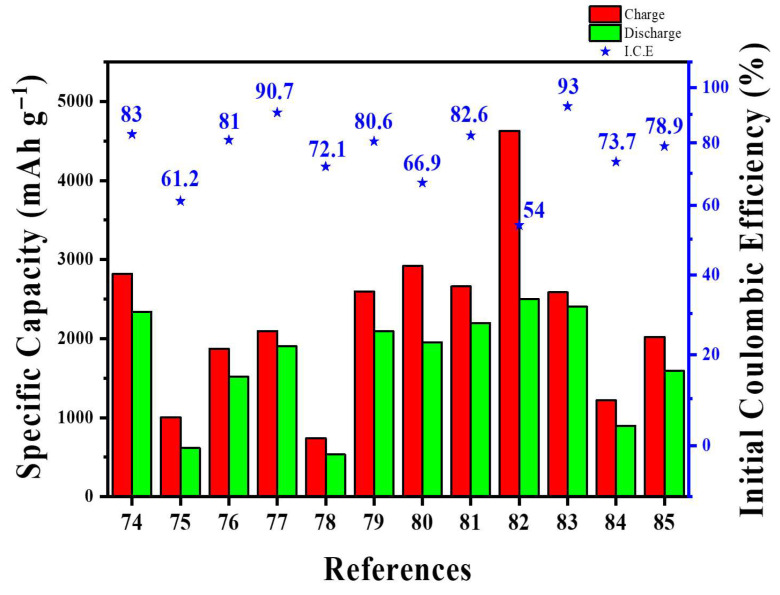
Direct quantitative comparison of various Si-graphene architectures in terms of key battery performance metrics.

From a scale-up perspective, graphene-based Si anodes face a trade-off between structural precision and manufacturing simplicity. CVD-grown multilayer graphene directly on Si nanoparticles or 3D graphene scaffolds achieved high volumetric energy density and long cycle life [74,76], but these structures required high-temperature gas-phase reactors and batch processing methods that are difficult to integrate with low-cost, high-throughput powder production. In contrast, methods such as aqueous spray-dried SiO_x_/C/QrGO, functionalization-assisted ball-milled Si/graphene composites, and graphene-coated SiO_x_/graphite blends were prepared by mixing, spray-drying, or ball-milling, then processed by standard slurry casting. They delivered areal capacities ≥3 mAh cm^−2^ and have been tested in pouch cells with NCM cathodes [75,78,80,85]. These slurry-compatible approaches lowered capital barriers but posed new challenges: controlling graphene sheet size and defect density is critical to prevent excessive slurry viscosity, poor tap density, or unstable SEI formation. Furthermore, the oxidative/reductive chemistries involved in graphene oxide processing presented environmental and wastewater management issues [86,87,88]. Therefore, to provide the most viable pathway from laboratory research to industrial application, a balance between material and process design should be achieved, encompassing simple synthesis of graphene and Si particles, the development of elaborate hybrid structures, and control of battery electrode manufacturing. Table 4 and Figure 10 provide the summary and quantitative comparison of various Si-graphene architectures in terms of key battery performance metrics.

## 3. Applications of Other Carbon Materials

To address the significant volume expansion and brittleness of Si anodes, carbon-based hybridization—especially carbon nanofiber networks and pitch-derived carbon coatings/supports—has become an effective design strategy. This chapter examines the CNF- and pitch-based approaches through a structure–process–performance perspective. It also discusses practical considerations for scaling up, including binder- and current-collector-free fabrication, electrolyte wetting, electrode-thickness management at high loadings, coating uniformity, and SEI/electrode co-optimization under thermal and high-rate conditions.

A binder-free, free-standing Si anode was created from Si encapsulated in hollow carbon nanospheres, assembled into microspheres, and cross-linked by N-doped carbon fibers (SHCM/NCF). This design tackled the issues of electronic conductivity and mechanical instability in Si anodes [89]. The hollow carbon shells provided space inside to buffer Si expansion, while evenly confining Si nanodots, which reduced excessive SEI growth. The architecture was made by creating Stöber-derived SiO_2_ cores with a resorcinol–formaldehyde (RF) shell, carbonizing the RF, converting SiO_2_ to Si through magnesiothermic reduction followed by acid washing, and then electrospinning the Si@hollow-carbon (SHC) with PAN/PVP in DMF into mats that were stabilized (≈250 °C) and carbonized (≈700 °C) to form N-doped cross-linking fibers. Electrochemically, the SHCM/NCF anode demonstrated excellent rate capability (≈1960→800 mAh g^−1^ from 0.2 to 3.2 A g^−1^) and durable cycling (≈1442 mAh g^−1^ after 800 cycles at 1 A g^−1^, ≈86% retention, CE > 98%), and delivered ≈450 mAh g^−1^ after 200 cycles at 0.5 A g^−1^ in LiCoO_2_ full cells. The authors also highlighted the processing and cost benefits of a binder-free flexible electrode, the reduced charge-transfer resistance shown by EIS, and strong mechanical flexibility (such as LED lighting when bent), emphasizing the practical potential of CNF-cross-linked hollow-carbon/Si architectures. Additionally, a binder- and collector-free paper anode with a loading level of 1.0–1.8 mg cm^−2^ and a Si fraction of 0.55 had a theoretical areal capacity of 2.31–4.16 mAh cm^−2^ (Qtheo = 4200 mAh g^−1^) or 1.97–3.54 mAh cm^−2^ (Qtheo = 3579 mAh g^−1^), meeting the threshold at the upper end of the practical areal loading range (≥3 mAh cm^−2^).

**Figure 11:**
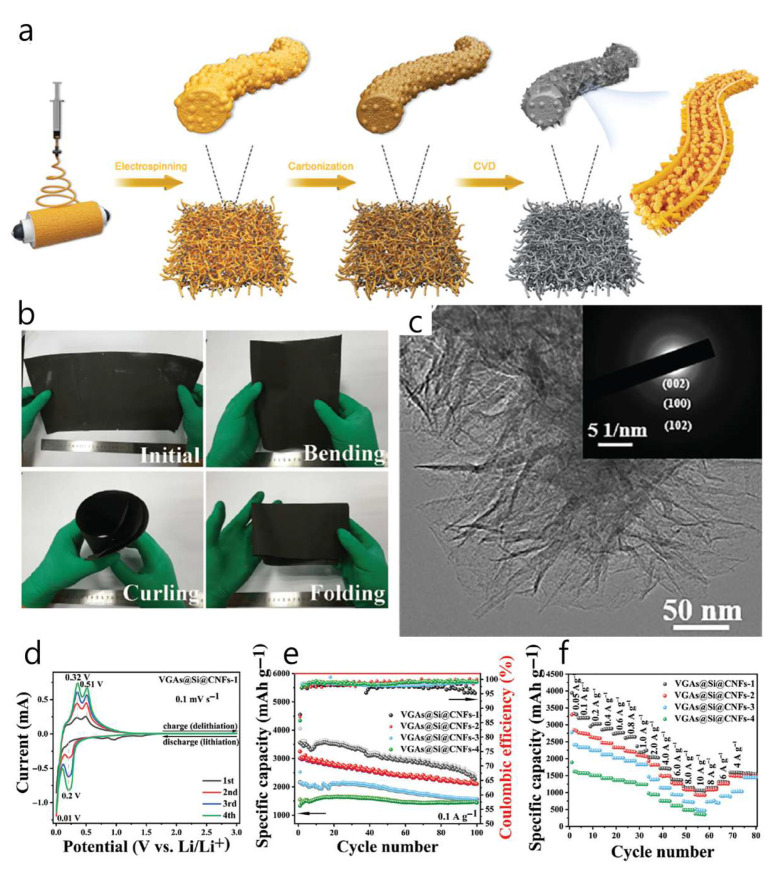
(**a**) Schematic illustration of the fabrication process of highly flexible VGAs@Si@CNFs electrodes through the electrospinning and CVD methods. (**b**) Photographs and demonstration of the flexible characteristic of VGAs@Si@CNFs. (**c**) HRTEM image of VGAs@Si@CNFs. (**d**) The initial four CV curves of the VGAs@Si@CNFs-1 electrode at 0.1 mV s^−1^. (**e**) The cycling stability under 0.1 mA g^−1^. (**f**) Rate capability of VGAs@Si@CNFs-X (X = 1, 2, 3, 4). (Reproduced with permission from [90], copyright 2022, Wiley-VCH GmbH).

It was reported that a free-standing, binder-free Si anode, in which ultra-high-content Si nanoparticles (>90 wt%) are embedded in carbon nanofibers and conformally bridged by nitrogen/oxygen-codoped vertical graphene arrays (VGAs@Si@CNFs), overcomes slow kinetics and mechanical instability in Si anodes [90]. The 3D all-carbon architecture, consisting of highly graphitized CNF networks interconnected with defect-rich VGAs, provides continuous electron/ion pathways and a mechanically strong framework that limits Si volume change. Meanwhile, the N/O codopants introduce abundant active sites and improve electrolyte wettability. Fabrication involves electrospinning of Si@PAN, followed by tension-assisted stabilization (~260 °C) and carbonization (~1000 °C) to produce Si@CNFs. Subsequently, vertical graphene is grown on the fibers via thermal CVD (using ethanol as carbon source), controlling the nanosheet height (≈50–300 nm within 3 h) (Figure 11). Electrochemically, the electrode delivers a high specific capacity of ≈3619.5 mAh g^−1^ at 0.05 A g^−1^, shows excellent long-term cycling with ≈1093.1 mAh g^−1^ after 1500 cycles at 8 A g^−1^, and exhibits strong rate capability. Full-cell tests against LFP/LCO demonstrate practical feasibility. This work introduces a novel, controllable carbon-coating method, where Si–CNF composite fibers are first fabricated by electrospinning a Si/carbon-precursor polymer blend followed by carbonization, and vertical graphene arrays are subsequently grown via CVD, resulting in outstanding electrochemical performance.

A free-standing Si-rich anode with a nanofiber-in-microfiber carbon/Si structure was developed using coaxial electrospinning (core of PVA + Si; sheath of PAN), designed to achieve high Si loading alongside good mechanical strength and conductivity [91]. Its architecture features short, branched Si-rich nanofibers wrapped in a thin PVA-derived carbon layer, embedded within flexible PAN-based carbon microfibers, creating continuous electron/ion pathways, integrated 3D current collectors, and voids to cushion Si volume fluctuations. The electrode was fabricated via coaxial electrospinning, stabilized at about 280 °C, then carbonized at roughly 700 °C to yield carbon/Si composite mats suitable for direct application as collector-free anodes. Electrochemical tests with 40 wt% Si showed an capacity of approximately 900 mAh g^−1^, maintaining about 90% of capacity from cycle 50 to 250, and demonstrated quick coulombic efficiency recovery to nearly 100% after initial SEI formation. Full cells paired with LiMn_2_O_4_ delivered stable charge/discharge performance (~90 mAh g^−1^ at 0.2 C). The study emphasizes that the PAN shell prevents Si oxidation during processing, that meso/macro-voids around Si are crucial for preventing dead-Si formation and sustaining conductivity, and that keeping Si content at or below 40 wt% avoids the heterogeneous pulverization seen at 50 wt% or higher. Although coaxial electrospinning offers manufacturing benefits, such as producing binder- and collector-free, integrated 3D current collection mats, the relatively modest first-cycle capacity (~1100 mAh g^−1^) and less-than-ideal initial coulombic efficiency (~70%) limit its immediate application in LIBs and warrant further optimization.

Finally, we discuss pitch-based silicon hybrids. Unlike other nanocarbon materials, pitch is a by-product of petroleum refining and has traditionally been used as a precursor for synthetic graphite and carbon fibers [92,93]. More recently, it has also been actively explored as a protective coating for Si anodes. A waste-to-value route was reported that re-agglomerated fine natural graphite via spray drying with CMC/citric acid [94]. The resulting agglomerates were then coated with petroleum pitch and heat-treated in either a one-step or two-step process, yielding battery-grade, mechanically robust secondary particles. The resulting quasi-spherical agglomerates showed smoother, pitch-sealed surfaces with reduced specific surface area/microporosity (<10 nm pores covered), and retained structural integrity under calendaring. Electrochemically, the pitch-coated electrodes delivered higher initial Coulombic efficiency (ICE ≈ 86% vs. 80% for raw fines), reached CE > 99% by cycle 3, and sustained approximately 95% capacity retention over 200 cycles (C/20→C/9 protocol). Rate capability was comparable to commercial natural graphite up to 1 C, with differential capacity indicating improved kinetics for pitch-coated samples. This study upcycles waste fine graphite generated during natural-graphite production into battery-ready micrographite with appropriate size and mechanical robustness via pitch coating. The approach provides a useful starting point for the pitch-based Si-coating strategies discussed next.

**Figure 12:**
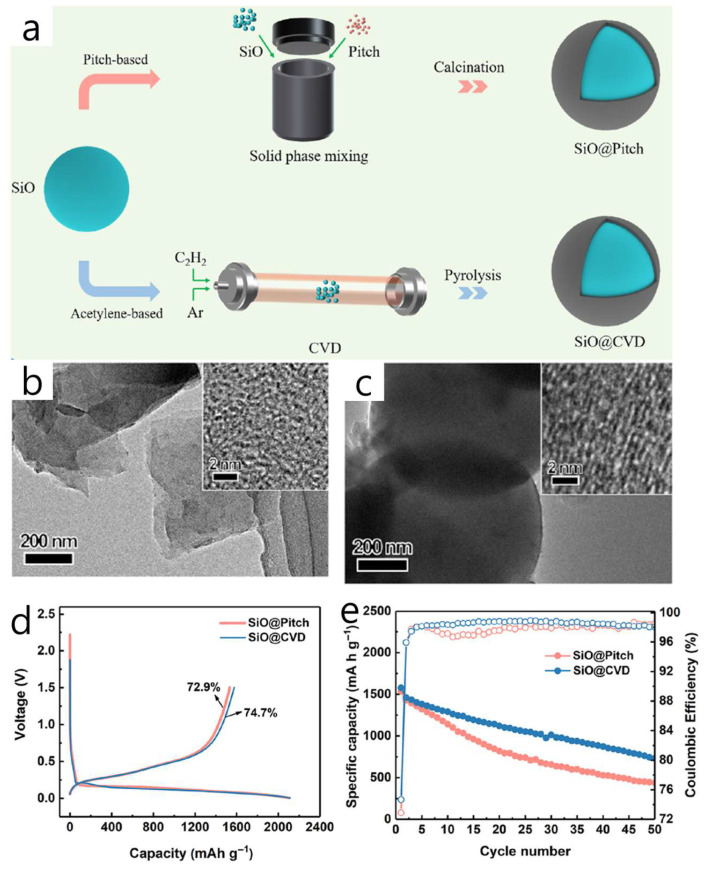
(**a**) Schematic illustration of the fabrication process for SiO@Pitch and SiO@CVD. HRTEM images of (**b**) C_pitch_, and (**c**) C_CVD_. (**d**) Voltage-capacity curves and (**e**) cycling performance of SiO@Pitch and SiO@CVD. The open circle represents Coulombic Efficiency. (Reproduced with permission from [95], copyright 2024, Elsevier).

The comparison between acetylene-CVD-derived and pitch-derived carbon coatings on SiO from coin half-cells to 6 Ah pouch full-cells showed that acetylene-based coatings achieved higher graphitization, better conductivity, and superior cycling stability (Figure 12a) [95]. The acetylene layer formed a more ordered, graphene-like lamellar carbon with a lower Raman ID/IG (~0.83 on SiO) and significantly lower powder resistivity (~0.5 Ω cm). This resulted in uniform, complete coverage that suppresses SEI growth. In contrast, pitch-derived carbon was more disordered (ID/IG ~ 0.98), much less conductive (~87.6 Ω cm), and non-uniform according to EDS/XPS, with thicker SEI after cycling (Figure 12b,c). SiO@CVD was produced in a rotary furnace at 900 °C for 2 h under 10 vol% C_2_H_2_/Ar, while SiO@Pitch was created by solid-phase mixing followed by heat treatments at 300 °C (1 h) and 900 °C (2 h). Full-cell tests used graphite-blended anodes (SiO 6.5 wt%, designed reversible capacity 420 mAh g^−1^, N/P ≈ 1.05) against NCM811. Electrochemically, SiO@CVD showed slightly higher initial charge capacity and ICE (≈1576 mAh g^−1^/74.7%) compared to SiO@Pitch (≈1534 mAh g^−1^/72.9%), along with lower Rct, larger DLi^+^, and much better cycling performance (≈732 vs. 437 mAh g^−1^ after 50 cycles at 0.2 C) (Figure 12d,e). In 6 Ah pouch cells, CVD-coated SiO retained capacity better at 25 °C and 45 °C, while performance was comparable at −10 °C. In summary, the acetylene-derived carbon coating demonstrated more uniform coverage and a higher degree of graphitization than pitch-derived layers, resulting in superior cycling stability and rate capability. However, because pitch properties vary significantly depending on feedstock and post-carbonization conditions, further research is necessary to evaluate its potential as a Si-coating precursor.

An investigation was conducted into how the macrostructure of pitch-derived carbon supports, engineered by KOH versus NaOH activation, influences Si loading during silane CVD and, consequently, the cycling stability of C/Si_x_/C anodes [96]. Porous supports displayed distinct void and crack morphologies (rounded ~µm voids for KOH, wider slit-like cracks > 1 µm for NaOH) that facilitated Si deposition within the carbon framework, buffered Si volume changes, and limited SEI growth compared with nonporous carbon. The composites were made in three steps: (i) activate petroleum pitch at 800 °C under N_2_ (KOH/pitch = 0.5 or NaOH/pitch = 2) to produce carbon supports; (ii) deposit Si via SiH_4_ CVD at 500 °C (1–2 h) to form Si_x_/C; and (iii) apply a conductive, robust carbon overlayer by CH_4_ CVD at 900 °C (1 h) to obtain C/Si_x_/C. Electrochemically, C/Si_x_/C produced on porous supports demonstrated higher capacity retention and stability than nonporous controls, with rate-capability trends aligning with internal Si deposition and improved Li^+^ diffusion coefficients. NaOH-activated, crack-rich carbons provided the most uniform internal Si distribution and the smallest volume expansion. While many studies have coated Si particles with one or two layers of carbon, this study prepared SiNPs between activated-carbon supports and then over-coated them with carbon, creating a distinctive C–Si–C architecture. By examining how the carbon-scaffold macrostructure affects Si loading, morphology, and cycling stability, the study offers a systematic framework for designing next-generation Si–carbon composite anodes.

Carbon coatings have been shown to reduce Si expansion but face material-level limitations: overly thick or uneven layers add dead weight, highly graphitized shells are expensive to produce, and pitch-derived films can be chemically heterogeneous and prone to cracking. Recently, to address these issues at the material level, studies have introduced hybrid interphases on Si particles by stacking self-healing polymers with carbon coatings [97]. Additionally, process-level approaches such as low-temperature CVD, plasma or laser processing, and pitch-chemistry tuning have been employed to improve the uniformity of the carbon layer, the degree of graphitization, and ionic/electronic conductivity [98].

**Figure 13:**
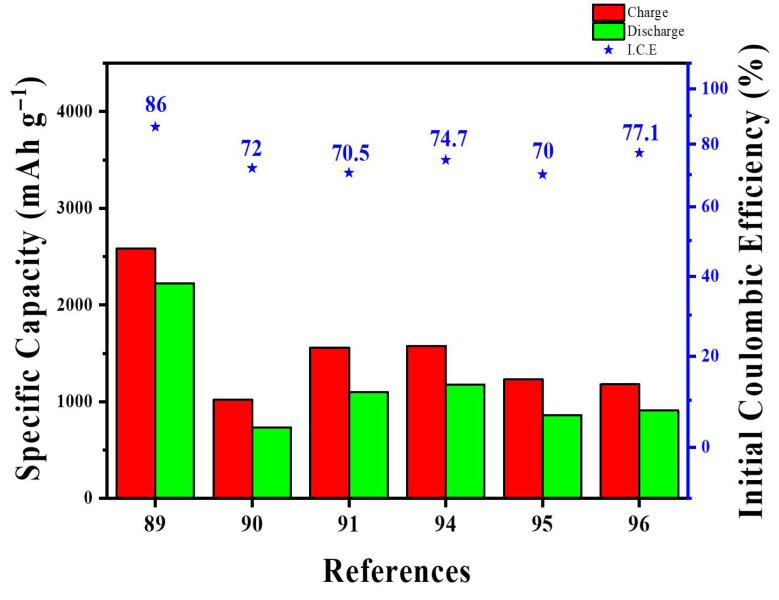
Direct quantitative comparison of various Si-carbon architectures in terms of key battery performance metrics.

For CNFs and pitch-derived carbons, scalability trade-offs are greatly affected by the choice of precursor and thermal history. Electrospun CNF networks and hollow-carbon-sphere/N-doped-CNF papers provided excellent cycling durability and binder-free designs [89,90,91]. However, they required multiple electrospinning steps, oxidative stabilization, and high-temperature graphitization, which are energy-intensive and currently restrict production volume. Conversely, pitch- and petroleum-derived carbons produced through KOH/NaOH activation, spray-drying of waste graphite fines, or pitch coating on SiO used inexpensive feedstocks and processing methods already familiar to synthetic-graphite producers [94,95,96]. Techno-economic and life-cycle analyses of pitch-based carbon fibers and MCMB-type carbons indicate these methods can be cost-competitive per kilogram when yield and furnace efficiency are optimized. Still, they face practical challenges such as alkali neutralization, VOC management, and precise control of mesophase formation to ensure consistent tap density and rate performance [37,38]. Therefore, the most practical CNF/pitch-derived structures for Si anodes are those that rely on existing graphite or carbon-fiber manufacturing lines and add incremental modifications, such as activation, spray-drying, or thin carbon coatings, rather than specialized, low-throughput nanofiber production. Table 5 and Figure 13 provide the summary and quantitative comparison of various Si-carbon architectures in terms of key battery performance metrics.

## 4. Commercialization Status and Production Technologies

Silicon-based anodes have advanced beyond the lab into pilot and manufacturing phases at multiple companies. Sila is scaling the delivery of its nano-composite silicon powder (“Titan Silicon”) with the construction of the Moses Lake, Washington, facility (expected to be finished in Q1 2025), along with a supply agreement with Panasonic for EV cells and collaborations with major OEMs [99]. Amprius is shipping CVD-grown silicon nanowire anode cells to aerospace customers from its U.S. pilot line and has announced 450 Wh kg^−1^-class SiCore™ cells with a near-term pathway to mass production [100]. Group14 is expanding production of Si–C composite (SCC55^®^) materials in the U.S. and Korea, enhancing global supply through additional investment and ownership consolidation of its Korean site [101]. OneD Battery Sciences, working with GM, is developing a process that grows silicon nanowires directly on graphite (SINANODE^®^), emphasizing compatibility with existing graphite-electrode manufacturing lines [102]. Large established anode suppliers like BTR and Shanshan are already delivering SiO_x_/C-type spherical particles produced via spray-drying and carbonization to multiple customers, demonstrating high ICE and pressability at the electrode level [103]. Nexeon is constructing a commercial silicon-anode materials plant in Gunsan, Korea, with deliveries to Panasonic expected from 2025 and secured long-term monosilane supply [104].

From a manufacturing perspective, common industrial methods can be grouped into: (i) spray-dried Si/SiO_x_–C microspheres designed for high tap density, strong calendaring, and stable slurry rheology; (ii) CVD/plasma growth of silicon nanostructures aiming for ultra-high energy density and high-rate performance; (iii) hierarchical carbon coatings—often pitch or resin derived—that enhance electronic percolation and stabilize the SEI; and (iv) direct growth of silicon nanowires on graphite to reduce disruption to existing lines [99,100,101,102,103,104]. Successful cell integration at a commercial scale typically involves co-optimizing pre-lithiation (e.g., SLMP), binder systems (PAA/CMC/SBR and high-solids slurries), and increasingly, dry-electrode processing to improve initial coulombic efficiency (ICE) and limit through-thickness expansion. Industrial targets include areal capacity ≥ 3 mAh cm^−2^, calendered electrode density ≥ 1.6 g cm^−3^, as well as minimized expansion and gas evolution, with proven calendar life at 25–45 °C.

Overall, commercialization progresses along a spectrum from SiO_x_/graphite blends (high reliability and ICE) to Si–C composites (balanced capacity and rate), and onward to nanowire/high-silicon-fraction designs (maximum specific energy), with each approach tailored to its target market. The carbon-based strategies reviewed in this work, including MWCNT/SWCNT networks, graphene scaffolds, carbon nanofibers, and pitch-derived carbon coatings, are directly compatible with these industrial processes. They enable the simultaneous achievement of high tap density, low expansion, and high ICE, all of which are essential for durable, energy-dense, and manufacturing-ready silicon anodes.

## 5. Conclusions

This review has demonstrated that MWCNTs and SWCNTs form strong 1D conductive networks that support electron flow and reduce stress, though challenges such as dispersion, agglomeration, and scalability remain. Graphene, with its exceptional flexibility and high conductivity, offers versatile 2D structures that improve SEI stability and allow high tap density, but balancing layer thickness, defect levels, and cost-efficient processing remains necessary. CNFs have advantages in forming interconnected frameworks with strong mechanical strength, though their larger diameter may limit conductivity efficiency at low loadings. Pitch-based carbons, as abundant and affordable precursors, help produce uniform coatings and facilitate graphitization, but variations in chemical composition and graphitization remain a concern for reproducibility.

The most promising approach involves designing hierarchical and multifunctional hybrids that combine different forms of carbon to leverage their respective strengths [105,106]. For instance, pairing CNTs with graphene or CNFs can create percolated electron/ion pathways with strong mechanical flexibility, while pitch-derived carbons can act as scalable coatings or structural frameworks [107,108,109]. Based on these factors, the leading candidate for the Si–carbon architecture with the highest commercial readiness is a pitch coating on Si microparticles combined with SWCNTs. Pitch coating already benefits from extensive expertise, cost advantages, and process efficiency in synthetic-graphite manufacturing, while SWCNTs have advanced beyond mere proof-of-concept as additives to reduce Si expansion. Given the current high cost of SWCNTs, exploring the use of high-quality graphene as an alternative should be considered. Instead of chemically reduced graphene oxide, electrochemically exfoliated graphene—with controlled defect levels, doping, and structure—could counteract the limited crystallinity and conductivity of pitch-derived carbon coatings [110,111].

The choice of carbon architecture should also consider energy consumption, precursor costs, and environmental impacts associated with their production routes. SWCNTs and MWCNTs typically rely on high-temperature CVD or arc/plasma methods, leading to substantial energy loss and elevated manufacturing costs despite their effective percolation and mechanical buffering at low loadings. Graphene exhibits similar technical hurdles, including high-temperature growth and chemically reductive routes, whereas electrochemical exfoliation in aqueous media provides a more sustainable balance between conductivity, defect tunability, and process eco-friendliness. CNFs occupy an intermediate regime in which electrospinning, stabilization, and carbonization steps are scalable but still require careful thermal management. Pitch-derived carbons utilize low-cost precursors and mature synthetic graphite infrastructure, though their graphitization still requires significant heat input.

Taken together, these considerations suggest that commercially viable Si–carbon hybrids will likely use small but effective amounts of nanocarbons to provide percolation and stress relief, while maximizing the use of pitch-based or other low-energy, scalable carbon frameworks. Such designs can lower energy consumption and material costs per kWh while maintaining high tap density, ICE, and compatibility with scalable manufacturing processes. As industrial applications expand, the criteria for selecting carbon materials should include sustainability metrics, such as energy consumption, greenhouse gas emissions, and precursor availability, along with traditional electrochemical and mechanical performance.

Moreover, moving from laboratory prototypes to industrial adoption will require more focus on electrode-level metrics like tap density, areal capacity, electrolyte compatibility, and manufacturing compatibility with roll-to-roll processes. A practical electrode-level approach that connects lab-scale materials to manufacturable cells is suggested: (i) developing thin Si/SiO_x_–C layers with pitch-derived conformal shells or a minimal CNT/graphene fraction (≤0.5 wt%) to ensure high tap density and through-thickness percolation; (ii) co-optimizing binder chemistry (PAA/CMC/SBR or ion-conductive variants), calendaring, and electrolyte/additive formulations to stabilize the SEI and improve ICE; and (iii) exploring and considering various anode manufacturing processes that can replace traditional slurry-based methods, focusing on throughput, yield, and energy cost (Table 6). Specifically, short-term targets to ensure results are industry-relevant include an areal capacity ≥ 3.0 mAh cm^−2^, calendered density ≥ 1.6 g cm^−3^, ICE ≥ 90%, and through-thickness swelling ≤ 20% during 0.5 C/200-cycle testing at 25–45 °C. Long-term efforts should aim to develop hierarchical networks (CNT + graphene/CNF) on porous or yolk–shell Si/C to meet ≥3C/500-cycle testing requirements with controlled expansion.

In conclusion, carbon-based hybrids remain essential for developing high-performance Si anodes. By critically understanding the strengths and limitations of MWCNTs, SWCNTs, graphene, CNFs, and pitch, and by designing synergistic architectures that leverage their benefits, it is possible to envision scalable, durable, and energy-dense Si anodes for next-generation LIBs.

## Figures and Tables

**Table 1:** Detailed comparison of differences among various carbon materials.

Materials	Electrical Conductivity (S/cm)	Specific Surface Area (m^2^/g)	Defect Content (*I_D_*/*I_G_*)	Cost ($/kg)	Precursor Cost ($/ton)	Slurry Viscosity at 1~2 wt%, 0.1 S^−1^ (η, mPa·s)	Number of Steps (in Battery Mfg.)	Overall Energy Consumption (kWh/kg)	Scalability (Tons/Year)	Refs.
MWCNT	10~500	10~300	0.7~1.2	50~300	600–1000	2000~5000	5~6	150–500	10,000–50,000	[18,19,20,21]
SWCNT	1000~10,000	800~1300	0.01~0.1	1500~2000	1400–3300	>10,000	6~7	5000–20,000	100–200	[21,22,23,24]
Graphene	100~1500	300~1000	0.1 (CVD), 1.0–1.5 (rGO)	50~500	1000–3000	3000~7000	6~7	50–200 (rGO)	1000–5000 (rGO, GNP)	[25,26,27,28,29]
CNF	5~100	20~300	0.9~1.3	~25	200–500	500~1500	5~6	100–300	300–1000	[30,31,32]
Pitch-derived Carbon	10~800 (MCMB), 2000~10,000 (CF)	1~20 (MCMB), ~5 (CF)	1.0~1.8	10~80 (MCMB), 20~100 (CF)	700–1300	<500	7~9	100–120 (MCMB), 15–100 (CF)	100,000	[33,34,35,36,37,38]

**Table 2:** Summary of MWCNT-based strategies for structurally stable silicon anodes.

Key Features	Initial Capacity (mAh g^−1^)	ICE (%)	Electrode Loading (mg cm^−2^)	Cycling Stability (mAh g^−1^)	Retention Rate (%)	C-Rate Condition	Ref.
MWCNT@Si core–shell (magnesiothermic)	1547/786	51	N/R	520 after 70 cycles	N/R	0.4 A g^−1^	[39]
Si–MWCNT–C microspheres	1848/1228	66.4	N/R	~1050 after 60 cycles	N/R	0.2 A g^−1^	[40]
Si sputtered on MWCNT buckypaper	930.6/107.8	12	~1.8	193 μAh after 100 cycles	99.5	125 μA	[41]
Si/Ag/Porous-MWCNT (Si/Ag/PM)	2163/1707	78.9	~1.0	706 after 300 cycles	68.6	1.0 A g^−1^	[42]
Si–MWCNTs@FPC + SC (freeze-dried)	2300/1260	54.8	2.0	501 after 500 cycles	>99.5	1.0 A g^−1^	[43]
SiO_2_/Si/PAN + rGO:MWCNT (1:1)	3009/2889	96.0	~0.8	1513 after 100 cycles	N/R	0.1 C	[44]
Graphite blended MWCNT-implanted Si composite (Gr-MSC)	573/500	87.3	~4.5	492 after 50 cycles	98.4	0.5 C	[45]
Si/MWCNT tissue (sputtered)	≈1300/299	23	~2.0	39% after 100 cycles	39	0.07 mA cm^−2^	[46]
Si/MWCNT@C spray-dried microsphere	1466/1026.2	70	1.0–1.2	536.6 after 300 cycles	80.5	1 A g^−1^	[47]
Defect-engineered MWCNTs	1565.1/626.1	40	0.7–1.0	856 after 100 cycles; 88% after 500 cycles	88.2	0.1, 0.5 A g^−1^	[48]
Prelithiated MWCNT–Si/Gr with NCM 622 full cell	200.6/174.9	87.2	16	129.3 after 100 cycles (NCM 622 full cell)	73.9	0.5 C	[49]
MWCNT–Si@Ag@CN (recycled Si)	2672.7/1273.5	58.0	1.0–1.2	1077.7 after 100 cycles (0.1 A g^−1^); 580.0 after 100 cycles (0.5 A g^−1^)	97.8 (at 1 A g^−1^)	0.1, 0.5, 1 A g^−1^	[50]

**Table 3:** Summary of SWCNT-based strategies for structurally stable silicon anodes.

Key Features	Initial Capacity (mAh g^−1^)	ICE (%)	Electrode Loading (mg cm^−2^)	Cycling Stability (mAh g^−1^)	Retention Rate (%)	C-Rate Condition	Ref.
Binder-free SWCNT vs. MWCNT (SiO_x_@C)	1785/1455	81.5	~1.5–2.0	915.9 after 200 cycles; 658.8 after 500 cycles	N/R	1.0 A g^−1^	[54]
CNT/CNF additives replacing Super P	N/R	N/R	~1.0	~60% capacity retention after 45 cycles	~60	0.5 C	[55]
Fe–Si alloy/rGO + N–SWCNT (8 wt%)	1416/1224	86.4	2.75, 12.8 (NCM 811 full cell)	82.3% after 100 cycles; 65% in NCM 811 full cell after 200 cycles	82.3/65	0.1 C (half-cell), 0.5 C (NCM 811 full cell)	[56]
SWCNT in SiO/graphite (contact integrity)	N/R	N/R	N/R	>90% after 90 cycles	>90	0.1–0.5 C	[57]
PPBT/SWCNT@Si MPs (capping layer)	~2230/1895	85.0	~1.0	1894 after 300 cycles; ~150 in NCM 523 full cell with ~80% retention after 50 cycles	92/~80	2.0 A g^−1^ (half-cell), 0.2 C (full cell)	[58]
SiMP/SWCNT–COOH@PVA–PAA (self-standing)	2973/2868.5	96.5	1.5–2.0	1795.6 after 300 cycles; ~1000 after 1000 cycles	96.5	0.2 C (100 cycles), 1 C (1000 cycles)	[59]
Dual-layer Si@C@SWCNT	2337/2072	88.7	~2.0	1267.3 after 500 cycles	N/R	1.0 A g^−1^	[60]
Ultra-lean SWNT (1 wt%) in SiO_x_@C	600/491.4	81.9	N/R	474 in NCM 811 full cell after 400 cycles	493 Wh/kg with 81.7	0.5 A g^−1^ (in NCM 811 full cell)	[61]
Long vs. Short CNT (acupuncture effect)	SW-l: 1781/1386; SW-s: 1760/1360; MW-l: 1752/1352; MW-s: 1731/1314	75.8–77.9	1.6–1.8	SW-l: 989; MW-l: 883; SW-s: 786; MW-s: 712 (after 200 cycles)	~55–60	0.5 A g^−1^	[62]
Ultra-low CNT in Si–graphite with SC-NCM pouch cell	~1372.3/~1198	87.3	7.8 (anode), 22.0 (cathode)	94.6% after 100 cycles	94.6	pouch full cell, ~0.5 C	[63]

**Table 4:** Summary of graphene-based strategies for structurally stable silicon anodes.

Key Features	Initial Capacity (mAh g^−1^)	ICE (%)	Electrode Loading (mg cm^−2^)	Cycling Stability (mAh g^−1^)	Retention Rate (%)	C-Rate Condition	Ref.
Multilayer graphene on Si (CVD growth)	2820/2340	83	0.3–2.5	700 Wh/L after 200 cycles (LiCoO_2_ full cell)	72 in LiCoO_2_ full cell	0.5 C in LiCoO_2_ Full cell	[74]
Si/rGO/C porous microspheres	1005/615	61.2	1.5–1.8	602 after 500 cycles	75	0.4 A g^−1^	[75]
N-graphene@Si@silicate sandwich	1871/1516	81.0	0.085–0.308	817 after 10,000 cycles	N/R	5.0 C	[76]
Pressure-tuned Si@graphene	2096.9/1902.6	90.7	1.3	1730 after 150 cycles	82.6	300 mA g^−1^	[77]
Graphene-coated SiO_x_/graphite	~740/533	72.1	3.0–5.0	620 after 300 cycles	69	0.5 C	[78]
Si@graphene@TiO_2_ core–shell	2597.9/2094.5	80.6	1.0–1.2	1005.1 after 300 cycles	68	2.0 A g^−1^	[79]
Si@graphene (APTES ball-milled)	2919.4/1952.8	66.9	0.1–0.3	1151.5 after 1000 cycles	N/R	1.0 A g^−1^	[80]
Si with graphitic graphene shell	2660.1/2198	82.6	3.05	747 after 500 cycles	N/R	1.0 C	[81]
Si@C–graphene hydrogel	up to 2200/~2200	>99	0.8	2200 after 200 cycles	>99	C/5	[82]
Si@rGO(S) small-sheet shell	2586/2405	93.0	1.0–1.1	2018 after 100 cycles; ~78% after 150 cycles	78	0.1 C	[83]
3D-printed Si/rGO grid	1219.4/898.0	73.7	70 mg/mL	930.6 after 350 cycles	68.9	C/2	[84]
SiO_x_/C@QrGO core–shell	2023/1596	78.9	N/R	~87% after 100 cycles; 76% after 500 cycles (NCM 622 full cell)	87/ 76 in NCM 622 Full cell	0.5 C (half-cell), 1 C(NCM 622 full cell)	[85]

**Table 5:** Summary of other carbon material-based strategies for structurally stable silicon anodes.

Key Features	Initial Capacity (mAh g^−1^)	ICE (%)	Electrode Loading (mg cm^−2^)	Cycling Stability (mAh g^−1^)	Retention Rate (%)	C-Rate Condition	Ref.
Hollow C nanospheres + N-doped CNF (binder-free paper)	2583/2224	86	1.0–1.8	1442 after 800 cycles; 450 after 200 cycles (in LiCoO_2_ full cell)	86/ 50.5 in LiCoO_2_ full cell	1.0 A g^−1^ (half-cell), 0.5 A g^−1^ (LiCoO_2_ full cell)	[89]
Nitrogen/oxygen co-doped vertical graphene grown on Si-coated CNF	3233	~75	0.8–1.5	2390.5 after 100 cycles	73.9	0.1 A/g	[90]
Nanofiber-in-microfiber C/Si composite (40 wt% Si)	2458/1749	71.1	1.95	900 after 250 cycles	51.5	50 mA/g	[91]
Upcycled waste graphite fines via spray-drying and pitch coating	410/351	86.2	5.1	331 after 200 cycles	95.1	0.11 C	[94]
Pitch vs. acetylene-CVD carbon on SiO	2110/1576 (CVD); 2106/1534 (Pitch)	74.7 (CVD); 72.9 (Pitch)	2.5	732 (CVD); 437 (Pitch) after 50 cycles	46.5 (CVD); 28.5 (Pitch)	0.2 C (half-cell)	[95]
Pitch-derived porous C support to C/Si_x_/C (Na, K, PC)	N/R	78.8 (Na); 87.6 (K); 79.2 (PC)	1.5–2.0	N/R	69.5 (Na); 67.4 (K); 48.3 (PC) after 50 cycles	0.1 A g^−1^	[96]

**Table 6:** Comparison of anode manufacturing processes from the perspectives of throughput, yield, and energy cost.

Process	Throughput	Yield	Energy Cost vs. Slurry	Refs.
Conventional slurry	1–25 m/min	≥98%	1.0×	[112,113,114]
Spray drying	20–50 kg/h	≥95%	1.2–3.0 kWh/kg	[115,116]
Electrostatic assembly	~0.5–2 m/min	90–95%	lower (ambient, ovenless/low heat)	[117,118]
3D printing	0.3–2.4 m/min	70–90%	lower/medium (drying eliminated)	[119]
Electrospinning	0.02–2.6 g/h	80–90%	low–medium (low thermal load)	[120,121]
CVD/PECVD	0.06–2 m/min	90–99%	≫1.0× (vacuum/plasma)	[122,123,124]

## Data Availability

No new data were created or analyzed in this study. Data sharing is not applicable to this article.
